# The Second Oncogenic Hit Determines the Cell Fate of *ETV6-RUNX1* Positive Leukemia

**DOI:** 10.3389/fcell.2021.704591

**Published:** 2021-07-15

**Authors:** Guillermo Rodríguez-Hernández, Ana Casado-García, Marta Isidro-Hernández, Daniel Picard, Javier Raboso-Gallego, Silvia Alemán-Arteaga, Alberto Orfao, Oscar Blanco, Susana Riesco, Pablo Prieto-Matos, Francisco Javier García Criado, María Begoña García Cenador, Hanno Hock, Tariq Enver, Isidro Sanchez-Garcia, Carolina Vicente-Dueñas

**Affiliations:** ^1^Experimental Therapeutics and Translational Oncology Program, Instituto de Biología Molecular y Celular del Cáncer, Consejo Superior de Investigaciones Científicas/Universidad de Salamanca, Salamanca, Spain; ^2^Institute for Biomedical Research of Salamanca, Salamanca, Spain; ^3^Pediatric Oncology, Hematology and Clinical Immunology, Medical Faculty, Heinrich Heine University, Düsseldorf, Germany; ^4^Servicio de Citometría, Departamento de Medicina, CIBERONC (CB16/12/00400), and Instituto de Biología Molecular y Celular del Cáncer, Consejo Superior de Investigaciones Científicas/Universidad de Salamanca, Salamanca, Spain; ^5^Departamento de Anatomía Patológica, Universidad de Salamanca, Salamanca, Spain; ^6^Department of Pediatrics, Hospital Universitario de Salamanca, Salamanca, Spain; ^7^Departamento de Cirugía, Universidad de Salamanca, Salamanca, Spain; ^8^Cancer Center and Center for Regenerative Medicine, Massachusetts General Hospital, Harvard Medical School, and Harvard Stem Cell Institute, Boston, MA, United States; ^9^Department of Cancer Biology, UCL Cancer Institute, University College London, London, United Kingdom

**Keywords:** transcription factors, B-cell, somatic, germline, childhood leukemia, mouse models

## Abstract

ETV6-RUNX1 is almost exclusively associated with childhood B-cell acute lymphoblastic leukemia (B-ALL), but the consequences of ETV6-RUNX1 expression on cell lineage decisions during B-cell leukemogenesis are completely unknown. Clinically silent ETV6-RUNX1 preleukemic clones are frequently found in neonatal cord blood, but few carriers develop B-ALL as a result of secondary genetic alterations. The understanding of the mechanisms underlying the first transforming steps could greatly advance the development of non-toxic prophylactic interventions. Using genetic lineage tracing, we examined the capacity of ETV6-RUNX1 to instruct a malignant phenotype in the hematopoietic lineage by cell-specific Cre-mediated activation of ETV6-RUNX1 from the endogenous *Etv6* gene locus. Here we show that, while ETV6-RUNX1 has the propensity to trigger both T- and B-lymphoid malignancies, it is the second hit that determines tumor cell identity. To instigate leukemia, both oncogenic hits must place early in the development of hematopoietic/precursor cells, not in already committed B-cells. Depending on the nature of the second hit, the resulting B-ALLs presented distinct entities that were clearly separable based on their gene expression profiles. Our findings give a novel mechanistic insight into the early steps of ETV6-RUNX1+ B-ALL development and might have major implications for the potential development of ETV6-RUNX1+ B-ALL prevention strategies.

## Introduction

Despite the enormous increase in tumor biology knowledge over the last four decades, the prevention of cancer development is still a distant goal. A crucial step in the genesis of a tumor is the transition from a benign precancerous to a malignant cancerous state, but the mechanisms that establish and regulate aberrant cell identity, finally allowing tumor cells to emerge, are still largely unknown (Vicente-Duenas et al., 2018).

Childhood acute lymphoblastic leukemia (ALL) is characterized by recurrent preleukemic chromosomal translocations that usually occur before birth (Mori et al., 2002; Greaves, 2018). The translocation *t*(12;21) resulting in the formation of the chimeric transcription factor ETV6-RUNX1 that fuses *ETV6* (HGNC:3495) and *RUNX1* (HGNC:10471) genes is the most frequent structural aberration, accounting for 25% of B-cell precursor ALLs (B-ALL) (Mullighan, 2014; Pui et al., 2019). The fusion gene is present in 1–5% of newborn children, but the actual incidence of ETV6-RUNX1+ B-ALL is much lower (0.0001%) (Mori et al., 2002; Schafer et al., 2018). The ETV6-RUNX1 fusion gene thus confers a low risk of developing B-ALL and presents only a first oncogenic event (“first hit”) in the process of leukemogenesis. A preleukemic clone is created, which requires secondary postnatal genetic aberrations for leukemic transformation (Swaminathan et al., 2015; Greaves, 2018).

Early molecular events in ETV6-RUNX1-associated leukemogenesis have been elusive because these stages are usually not detected in children (Martin-Lorenzo et al., 2015; Rodriguez-Hernandez et al., 2017a,b; Greaves, 2018) and are difficult to deduce from already established tumors at diagnosis (Vicente-Duenas et al., 2018). We recently demonstrated in mice that natural infection exposure can trigger oncogenic secondary hits, leading to the transformation of susceptible ETV6-RUNX1+ preleukemic cells and the emergence of B-ALL (Rodriguez-Hernandez et al., 2017a). This genetic predisposition to B-ALL shapes an identifiable and distinct gut microbiome in mice that acts as a barrier for leukemia development (Vicente-Duenas et al., 2020). This first murine model of ETV6-RUNX1-associated B-ALL faithfully mimics the human disease and presents with similar secondary genetic hits, including recurrent disruption of *Kdm5c* (HGNC:11114), *Pax5* (HGNC:8619), and *Ebf1* HGNC:3126 (Rodriguez-Hernandez et al., 2017a).

ETV6-RUNX1+ B-ALL is considered a malignant counterpart to normal B-cell precursors because it is generally associated with B-ALL. It is further assumed that additional second hits driving B-cell leukemogenesis take place in precursor B-cells. This model proposes that the phenotype of the leukemic cells is comparable to normal B-cells transformed by a double hit. However, several studies have shown that, without proper functional lineage tracing, attempting to deduce the identity of the leukemic cell-of-origin from the phenotype of the established B-ALL can lead to false conclusions (Gilbertson, 2011). So far, efforts to model *ETV6-RUNX1* disease by expressing *ETV6-RUNX1* in B-cells [regulated by the immunoglobulin heavy chain enhancer/promoter (Andreasson et al., 2001) or the CD19 promoter (Kantner et al., 2013)] or in human pluripotent stem cell (hPSC)-derived B lineage progenitors (Boiers et al., 2018) have failed. The cell-of-origin and the role of the *ETV6-RUNX1* transcription factor in lineage organization during leukemogenesis have thus remained unclear. Here, using genetic engineering and *in vivo* lineage tracing, we have examined how ETV6-RUNX1 generates a B-ALL phenotype in mice.

## Materials and Methods

### Mouse Leukemia Models for *ETV6-RUNX1* B-ALL

**FIGURE 1:**
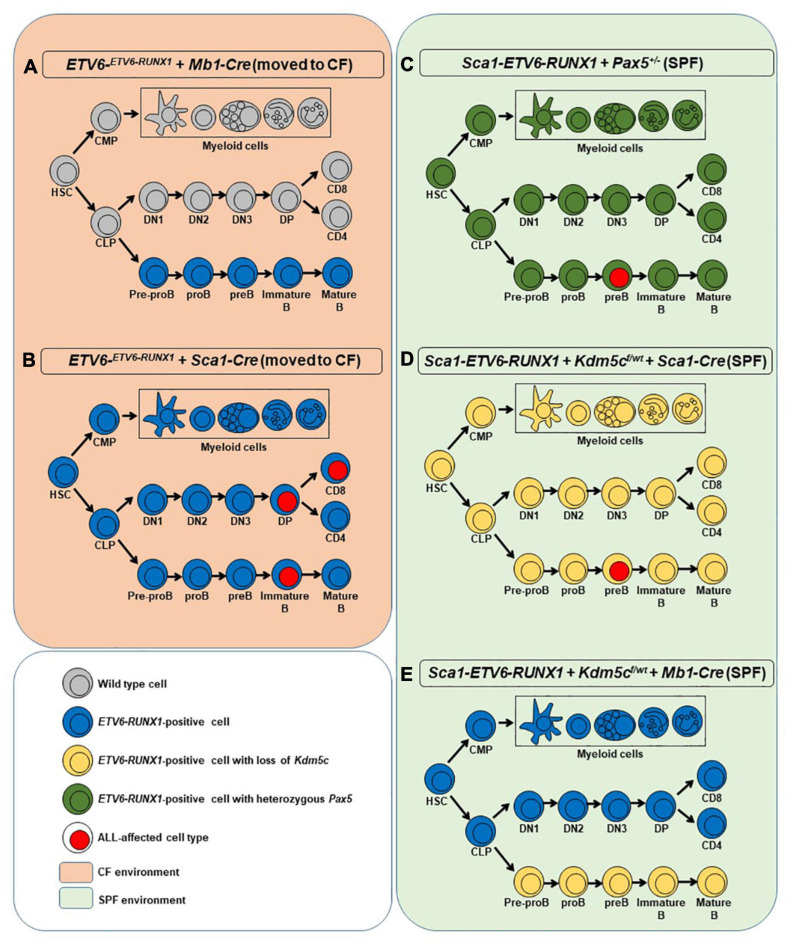
Overview of the mouse models used in the study. For each model, a simplified hematopoiesis is shown. The colors show at which stage *ETV6-RUNX1* (blue), the loss of *Kdm5c* (yellow), or the heterozygous loss of Pax5 (green) is introduced and which cells carry these aberrations. Cell types with surface markers identified from leukemic cells are marked with a red nucleus. The orange background on the left represents the two mouse models that were moved to the conventional facility where they were exposed to common pathogens **(A,B)**. In such conditions, only the introduction of *ETV6-RUNX1* at the HSC/progenitor stage led to ALL **(B)**. The green background on the right denotes the specific pathogen-free facility environment where the models in which the secondary hits were introduced were kept instead of exposing them to infectious stimuli **(C–E)**. Only if the secondary hit was introduced at HSC/progenitor stage ALL arose **(C,D)**. HSC, hematopoietic stem cell; CMP, common myeloid progenitor; CLP, common lymphoid progenitor; DN1-3, double-negative T cell progenitors 1-3; DP, double-positive T cell progenitor; CD8/CD4, single-positive naïve T cells.

*ETV6-^*ETV*6–RUNX1^*mice (Schindler et al., 2009) were bred with *Mb1−Cre* (Hobeika et al., 2006) or *Sca1−Cre* (Mainardi et al., 2014) to generate *ETV6-^*ETV*6–RUNX1^* + *Mb1-Cre* or *ETV6-^*ETV*6–RUNX1^* + *Sca1-Cre* mice, respectively. These *ETV6-^*ETV*6–RUNX1^* + *Mb1-Cre* and *ETV6-^*ETV*6–RUNX1^* + *Sca1-Cre* mice were born and kept at the specific pathogen-free (SPF) facility until exposed to a natural infectious environment as described (Figures 1A,B) (Rodriguez-Hernandez et al., 2017a). The *Kdm5c*^*f/wt*^ line was obtained from the European Mouse Mutant Archive (EMMA) public repository (strain name: C57BL/6N-A<tm1brdKdm5c<tm1c(EUCOMM)Hmgu>/Ics; strain ID: EM:06928). The Sca1-*ETV6-RUNX1* mouse model (Rodriguez-Hernandez et al., 2017a) was bred with *Kdm5c^*f/wt*^* + *Mb1-Cre* or *Kdm5c^*f/wt*^* + *Sca1-Cre* to generate Sca1-*ETV6-RUNX1* + *Kdm5c^*f/wt*^* + *Mb1-Cre* or Sca1-*ETV6-RUNX1* + *Kdm5c^*f/wt*^* + *Sca1-Cre*, respectively. The Sca1-*ETV6-RUNX1* mouse model (Rodriguez-Hernandez et al., 2017a) was bred with *Pax5-het* mice (Urbanek et al., 1994) to obtain Sca1-*ETV6-RUNX1* + *Pax5-het* mice (Figure 1C). Sca1-*ETV6-RUNX1* + *Kdm5c^*f/wt*^* + *Mb1-Cre*, Sca1-*ETV6-RUNX1* + *Kdm5c^*f/wt*^* + *Sca1-Cre*, and Sca1-*ETV6-RUNX1* + *Pax5-het* mice were born and kept at the SPF facility, where leukemia development was monitored (Figures 1C–E). The genotype of the mice was assessed following provider protocol and an example of Kdm5c allele deletion, as shown in Supplementary Figures 1, 2. All animal works were conducted in accordance with national and international guidelines on animal care and approved by the Bioethics Committee of the University of Salamanca and the Bioethics Subcommittee of Consejo Superior de Investigaciones Científicas under the approved project license (number 186). The study includes both male and female mice. There were no mice excluded from any experimental group. The housing environmental conditions were a temperature of 21 ± 2°C, humidity of 55 ± 10%, and a 12:12 light/dark cycle. The animals were housed in the SPF facility in individually ventilated cages and in conventional facility housing in conventional mouse cages (32 × 20 × 13.5 cm) with a maximum of five animals per cage. The mice had access to mice maintenance food (LABDIET PICOLAB SELECT; RODENT DIET 50 IF/6F IRRADIATED 5V5R; 3002906-203) and water *ad libitum*. Environmental enrichment included red-tinted Techniplast Mouse House and rodent roll. During housing, the animals were monitored daily for health status. The experiments were not blinded. The sample size of the experimental groups was based on the expected incidence of leukemia development as described in previous studies (Schindler et al., 2009; Martin-Lorenzo et al., 2015; Rodriguez-Hernandez et al., 2017a) and approved by the Bioethics Committee of the University of Salamanca under an approved project license (number 186). To monitor the development of the disease, blood samples were collected every 2 months by submandibular bleeding of mice using a single-use lancet without the use of anesthesia. Upon clinical manifestations of the disease, the mice were subjected to euthanasia and subjected to standard necropsy procedures. All major organs were examined under a dissecting microscope. Tissue samples were taken from homogenous portions of the resected organ and fixed immediately after excision. Differences in the survival of transgenic and control *WT* mice were analyzed using the log−rank (Mantel–Cox) test.

All sections of this report adhere to the ARRIVE Guidelines for reporting animal research (Kilkenny et al., 2010). The ARRIVE Essential 10 guidelines checklist is included in Checklist S1 as Supplementary Material (Supplementary Data Sheet 2).

### FACS Analysis

Nucleated cells were obtained from total mouse bone marrow (flushed from the long bones), peripheral blood, thymus, or spleen. To prepare cells for flow cytometry, contaminated red blood cells were lysed with red cell lysis buffer, and the remaining cells were then washed in phosphate-buffered saline (PBS) with 1% fetal calf serum (FCS). After staining, all cells were washed once in PBS and were resuspended in PBS with 1% FCS containing 10 μg/ml propidium iodide (PI) to allow dead cells to be excluded from both analyses and sorting procedures. The samples and the data were acquired in an AccuriC6 Flow Cytometer and analyzed using FlowJo software. The specific fluorescence of FITC, PE, PI, and APC excited at 488 nm (0.4 W) and 633 nm (30 mW). Known forward and orthogonal light-scattering properties of mouse cells were used to establish gates. Non-specific antibody binding was suppressed by preincubation of cells with CD16/CD32 Fc-block solution (BD Biosciences). For each analysis, a total of at least 50,000 viable (PI-) cells were assessed.

The following antibodies were used for flow cytometry: anti-B220 (RA3-6B2), CD3ε (145-2C11), CD4 (RM4-5, 1:250), CD8a (53-6.7, 1:250), CD11b/Mac1 (M1/70, 1:200), CD19 (1D3), CD117/c-Kit (2B8, 1:200), Ly-6G/Gr1 (RB6-8C5), IgM (RMM-1), and CD25 (PC61, 1:500) antibodies. All antibodies were purchased from either BD Biosciences or Biolegend. All antibodies were used at a 1:100 dilution unless otherwise indicated.

### Histology

The animals were subjected to euthanasia by cervical dislocation; tissue samples were formalin-fixed and included in paraffin. Pathology assessment was performed on hematoxylin–eosin-stained sections under the supervision of Dr. OB, an expert pathologist at the Salamanca University Hospital.

### Western Blot

Fluorescence-activated cell sorting (FACS)-sorted B-cells or cultured REH (cell line carrying ETV6-RUNX1 fusion) were washed in PBS, pelleted, re-suspended in cold radioimmunoprecipitation assay (RIPA) buffer (50 mM Tris, pH 8.0, 150 mM NaCl, 1% Igepal CA-630, 0.5% Na-deoxycholate, and 0.1% SDS) containing protease inhibitors (cOmplete Mini Protease Inhibitor Cocktail, Roche 11836153001), and incubated on ice for 10 min. The lysates were centrifuged at 20,000 × *g* for 10 min to pellet the insoluble fraction, the supernatants were removed, and the pellets were washed once in RIPA. The pellets were resuspended in RIPA and sonicated in a Bioruptor Pico (Diagenode) for 5 × 30 s. The samples were subjected to polyacrylamide gel electrophoresis and western blotting. ETV6-RUNX1 was detected with an antibody against RUNX1 (Abcam, ab23980, 1 ug/ml). β-Actin antibody was used as a loading control (C4: sc-47778, 1 ug/ml).

### V(D)J Recombination

Immunoglobulin rearrangements were amplified by PCR using published primers (Cobaleda et al., 2007) and listed in Supplementary Table 3. Briefly, PCRs were performed in a 50-μl reaction, in which 1 ul of genomic DNA was used as a template at a concentration of 200 ng/ul and 2 ul of primers at 0.1 ug/ul. One unit of DNA polymerase supplemented with its buffer and dNTP was added to the reaction (Cat: R001A; Takara). The cycling conditions consisted of initial heat activation at 95°C followed by 31–37 cycles of denaturation for 1 min at 95°C, annealing for 1 min at 65°C, and elongation for 1 min and 45 s at 72°C. This was followed by a final elongation for 10 min at 72°C.

### Mouse Exome Library Preparation and Next-Generation Sequencing

#### Sample Acquisition

The AllPrep DNA/RNA Mini Kit (Qiagen, Hilden, Germany) was used to purify DNA according to the manufacturer’s instructions.

#### Exome Library Preparation and Next-Generation Sequencing

Exome library preparation was performed using the Agilent SureSelectXT Mouse All Exon kit with modifications adapted from Fisher et al. (2011). Briefly, we added SPRI beads to the original protocol and reduced the size of the reaction to 0.5 μl to be able to use PCR tubes for subsequent steps. Furthermore, we reduced the volume for washing. We minimized sample loss and optimized sample processing by reducing sample handling. We therefore only added freshly prepared 20% PEG/2.5 M NaCl (Sigma) instead of eluting samples from the SPRI beads for library preparation. Targeted capture by hybridization to an RNA library was performed according to the manufacturer’s protocol. Purification and enrichment of the captured library were achieved by binding to MyOne Streptavidin T1 Dynabeads (Life Technologies) and off-bead PCR amplification in the linear range. Then, 2 × 100-bp sequencing with a 6-bp index read was performed using the TruSeq SBS Kit v3 on the HiSeq 2500 (Illumina).

#### Data Analysis

Fastq files were generated using bcltofastq 2.19.0.316 (Illumina). BWA version 0.7.12. (Li and Durbin, 2010) was used to align sequence data to the mouse reference genome (GRCm38.71). Conversion steps were carried out using Samtools 1.3.2 (Li and Durbin, 2009; Li et al., 2009) followed by removal of duplicate reads by Picard tools 2.0.1^1^. Local realignment around indels, single-nucleotide polymorphism calling, annotation, and recalibration were facilitated by GATK 3.5.0 (DePristo et al., 2011). Further details on the processing pipeline can be found online at https://github.com/sjanssen2/spike. Mouse dbSNP138 and dbSNP for the mouse strains used acted as training datasets for recalibration. The resulting variation calls were annotated by Variant Effect Predictor (McLaren et al., 2010) using the Ensembl database (v70) and imported into an in-house MariaDB database to facilitate automatic and manual annotation, reconciliation, and data analysis by complex database queries. Loss-of-function prediction scores for PolyPhen2 (Adzhubei et al., 2010) and SIFT (Kumar et al., 2009) were extracted from this Ensemble release.

Only entries with at least 9% difference in allele frequency between tumor and normal were kept for further analysis. Cancer-related genes were determined by translating the cancer gene consensus from COSMIC (Database issue) using ENSEMBL’s biomart.

Using the online available data from St. Jude Cloud PeCan^2^, we preselected a set of genes associated with human B- or T-ALL (Ma et al., 2018). Only mutated genes with at least 5% frequency in B- or T-ALL were included and used to filter mouse mutations that only occur in those.

### RNA Sequencing and Bioinformatics

Using the Truseq RNA sample preparation kit (Illumina), RNA sequencing libraries were generated using 500 ng of total RNA from blast cells obtained from the relevant mouse models, cells from healthy thymus, and FACs-sorted pro-B-cells as a control (WT) to prepare the barcoded libraries. The libraries were validated and quantified using DNA 1000 and high-sensitivity chips on a Bioanalyzer (Agilent, Böblingen, Germany); 7.5 pM denatured libraries were used as input into cBot (Illumina), followed by deep sequencing using HiSeq 2500 (Illumina) for 101 cycles, with an additional seven cycles for index reading.

Fastq files were imported into Partek Flow (Partek Incorporated, MO, United States). Quality analysis and quality control were performed on all reads to assess read quality and to determine the amount of trimming required (both ends: 13 5′ bases and one 3′ base). The trimmed reads were aligned against the GRCm38 mouse genome using the STAR v2.4.1d aligner. The unaligned reads were further processed using Bowtie 2 v2.2.5 aligner. The aligned reads were combined before quantifying the expression against the mmu ENSEMBL (release 95) database using the Partek expectation–maximization algorithm using the counts-per-million normalization. Genes with missing values or with a mean expression less than one were filtered out.

Finally, statistical gene set analysis was performed using *t*-tests to determine differential expression at the gene level (false discovery rate, *q* < 0.05; fold change ± 2). Partek flow default settings were used in all analyses.

### Statistical Analysis

Comparisons of survival curves estimated by Kaplan–Meier plots using Graph Pad Prism 5.0 were performed by the log-rank (Mantel–Cox) test. The differences between two sample groups were made using an unpaired *t*-test with GrapPad Prism 5.0 software. The level of significance was set at *p*-value < 0.05.

### Accession Numbers

The mouse RNA sequencing data have been deposited in NCBI’s Gene Expression Omnibus (GEO) and are accessible through GEO series accession number GSE141112^3^.

## Results

### *ETV6-RUNX1* Expression Restricted to the B-Cell Compartment Does Not Act as a First Oncogenic Hit Under Infection Exposure

**FIGURE 2:**
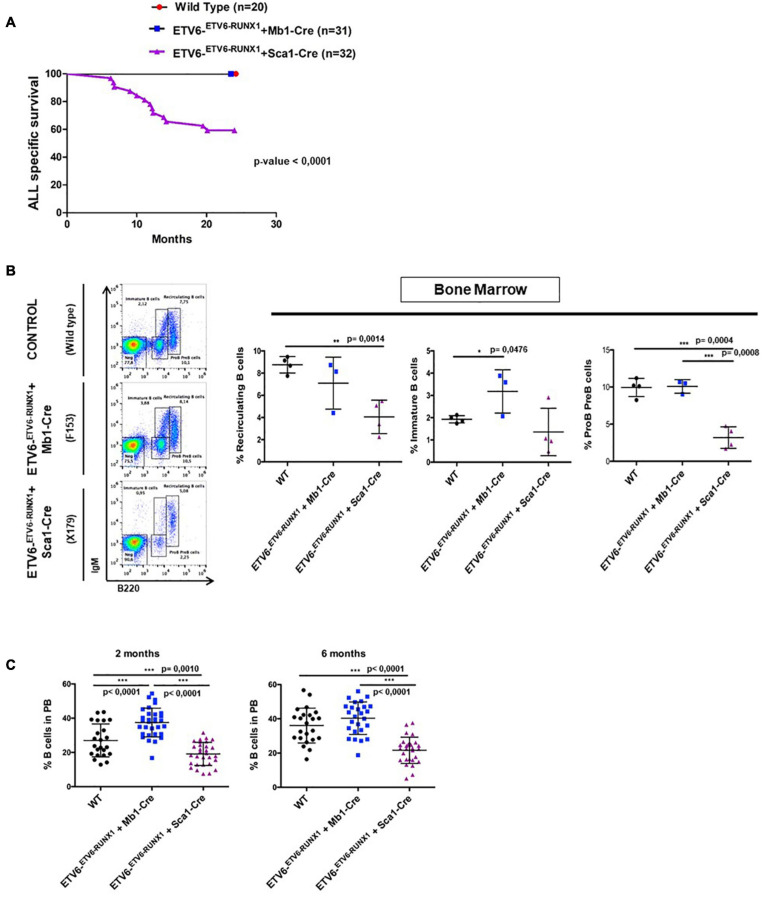
*ETV6-RUNX1* expression does not transform committed B-cells under natural infection exposure. **(A)** Leukemia-specific survival of ETV6-^*ETV*6–RUNX1^ + Mb1-Cre mice (blue points, *n* = 31) and ETV6-^*ETV*6–RUNX1^ + Sca1-Cre mice (purple line, *n* = 32), showing that the latter group had a significantly (log-rank test *p*-value < 0.0001) shortened lifespan compared to control littermate wild-type (WT) mice (red points, *n* = 20) as a result of B and T-ALL development. **(B)** B-cell development in young ETV6-^*ETV*6–RUNX1^ + Mb1-Cre mice (*n* = 3), ETV6-^*ETV*6–RUNX1^ + Sca1-Cre mice (*n* = 4), and age-matched (4 months old) WT mice (*n* = 4) as analyzed by flow cytometry. ETV6-^*ETV*6–RUNX1^ + Sca1-Cre mice show a significant decrease in pre-B/pro-B-cells (B220^*low*^ IgM^–^) and recirculating B-cells (B220^*high*^ IgM^+^) but not in immature B-cells (B220^*low*^ IgM^+^). A significant unpaired *t*-test *p*-value is indicated in each case. Error bars represent SD. **(C)** Flow cytometry analysis of peripheral blood B-cells in young ETV6-^*ETV*6–RUNX1^ + Mb1-Cre (*n* = 26–31), ETV6-^*ETV*6–RUNX1^ + Sca1-Cre (*n* = 28–30), and age-matched (2- and 6-month-old mice) WT mice (*n* = 23). ETV6-^*ETV*6–RUNX1^ + Sca1-Cre mice show a significant decrease in peripheral blood B-cells (B220^+^ IgM^±^).

The earliest steps of B-ALL development cannot be monitored in humans and have already concluded at the time of diagnosis. To clarify which hematopoietic cell compartment contributes to *ETV6-RUNX1*-associated transformation, we first tested whether *ETV6-RUNX1* expression restricted to the B-cell lineage can induce B-ALL. Since the impact of *ETV6-RUNX1* is likely dose dependent (Boiers et al., 2018), we used ETV6-^*ETV*6–RUNX1^ mice (Schindler et al., 2009), generated by conditional targeting of *Runx1* into the *Etv6* gene locus, thus placing *ETV6-RUNX1* gene expression under the control of the endogenous *Etv6* promoter. We targeted *ETV6-RUNX1* expression to the B-cell lineage by crossing the ETV6-^*ETV*6–*RUNX*1^ mice with an *Mb1-Cre* mouse strain (Hobeika et al., 2006). The generated strain (*ETV6-^*ETV*6–RUNX1^* + *Mb1-Cre*) expresses *ETV6-RUNX1* at the pro-B-cell level driven by Cre recombinase activation regulated by the promoter of the *Mb1* gene (encoding *Cd79a*, immunoglobulin-associated alpha chain, HGNC:1698) (Figure 1A). The resulting ETV6-RUNX1 protein levels were equivalent to ETV6-RUNX1 expression in the B-ALL cell line REH as confirmed by immunoblot analysis (Supplementary Figure 3). We then tested if B-ALL development can be provoked in *ETV6-^*ETV*6–RUNX1^* + *Mb1-Cre* mice through natural infection exposure (Figure 1A). To this end, cohorts of *ETV6-^*ETV*6–RUNX1^* + *Mb1-Cre* and control wild-type (WT) mice were born and kept in a SPF environment until transferred to a conventional facility providing a common infectious environment (including pathogens like murine norovirus, murine hepatitis virus, *Helicobacter* species, and *Trichomonas muris*) (Martin-Lorenzo et al., 2015; Rodriguez-Hernandez et al., 2017a). The mice were monitored for their entire lifespans (*n* = 31; observed for up to 2 years), but none of the mice developed leukemia under these conditions (Figure 2A). To study the long-term impact of *ETV6-RUNX1* on bone marrow (BM) lymphopoiesis, we characterized the developmental stages of B-cells in 4-month-old *ETV6-^*ETV*6–RUNX1^* + *Mb1-Cre* transgenic mice and age-matched WT controls in BM and peripheral blood (PB) by flow cytometry. B-cells from *ETV6-^*ETV*6–RUNX1^* + *Mb1-Cre* mice showed similar developmental patterns as B-cells from control littermates (Figure 2B), which indicated that the induction of *ETV6-RUNX1* at the pro-B-cell stage has no significant effect on B-cell development.

### Infection Exposure Can Trigger Leukemogenesis if *ETV6-RUNX1* Expression Is Initiated in HS/PCs

**FIGURE 3:**
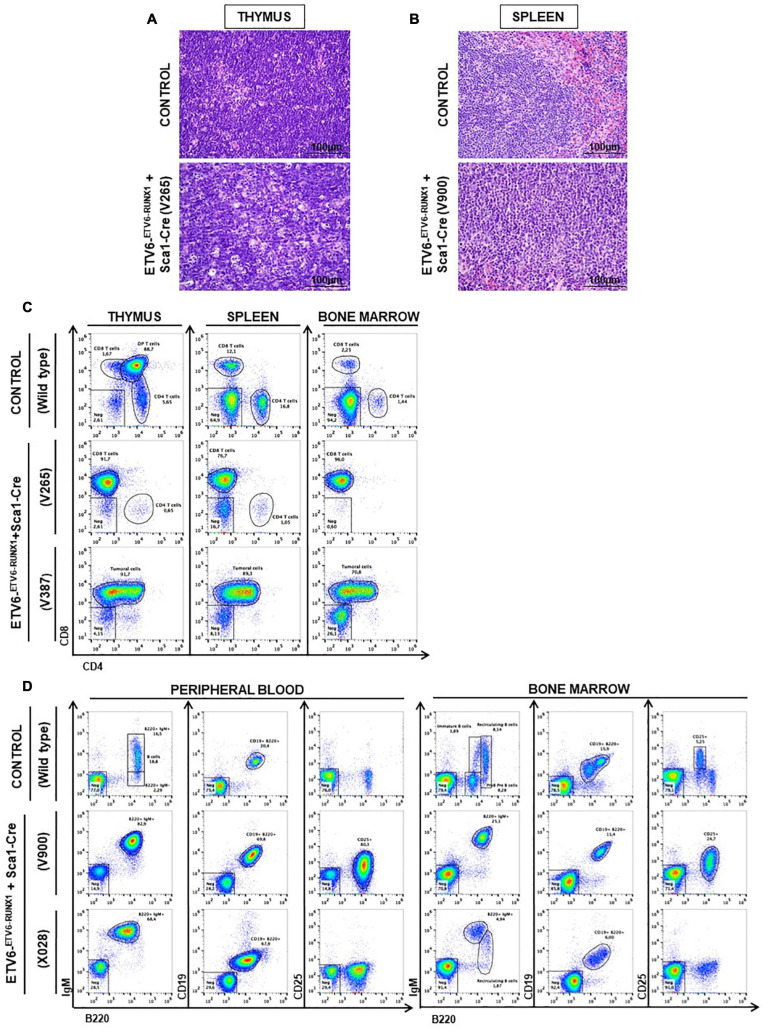
*ETV6-RUNX1* expression in hematopoietic stem or progenitor cells gives rise to lymphoid leukemia under natural infection exposure. **(A)** Hematoxylin and eosin staining showing the infiltration of the thymus in the ETV6-^*ETV*6–RUNX1^ + Sca1-Cre mice with T-ALL. The images were photographed at ×400 magnification. **(B)** Hematoxylin and eosin staining showing the infiltration of the spleen in the ETV6-^*ETV*6–RUNX1^ + Sca1-Cre mice with B-ALL. The images were photographed at ×400 magnification. **(C)** Flow cytometric analysis of T-cell subsets in the thymuses, spleen, and bone marrow of diseased ETV6-^*ETV*6–RUNX1^ + Sca1-Cre mice with T-ALL. Representative plots of cell subsets are shown. These exhibited the accumulation of DP-positive and/or CD8-positive tumoral T cells. Thymus, spleen, and bone marrow from a control littermate wild-type (WT) mouse are shown for reference. The flow cytometric images are representative of 32 mice analyzed. **(D)** Flow cytometric analysis of B-cell subsets in the peripheral blood (PB) and bone marrow (BM) of diseased ETV6-^*ETV*6–RUNX1^ + Sca1-Cre mice with B-ALL. Representative plots of the cell subsets are shown. These exhibited the accumulation of CD19^+^B220^+^IgM^+^CD25^±^ positive tumoral B-cells. The PB and BM from a control littermate WT mouse are shown for reference. Flow cytometric images are representative of 32 mice analyzed.

**FIGURE 4:**
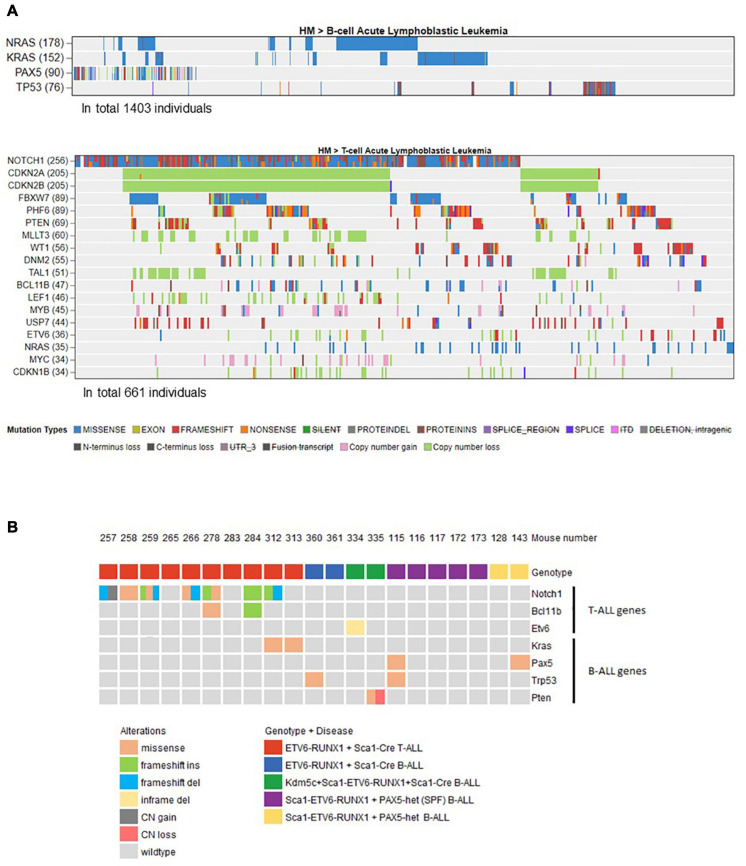
Overview of the mouse tumor mutations associated with human B- or T-ALL identified by whole-exome sequencing. **(A)** Using the online available data from St. Jude Cloud PeCan (https://pecan.stjude.cloud/), we preselected a set of genes associated with human B- (*n* = 1,403) or T-ALL (*n* = 661). Only mutated genes with at least 5% frequency (somatic mutations or copy number variations) in B- or T-ALL were included and used to filter mouse mutations that only occur in those. **(B)** Oncoprint representation of mouse mutations associated with human B- or T-ALL identified by whole-exome sequencing. Whole-exome sequencing analysis of tumor and control samples in *ETV6-^*ETV*6–RUNX1^* + *Sca1-Cre*, Sca1-*ETV6-RUNX1* + Kdm5c^*f/wt*^ + Sca1-Cre, and Sca1-*ETV6-RUNX1* + *Pax5-het* mice leukemias. Tumor DNA was derived from whole bone marrow in B-ALL samples, and in the case of T-ALL, either thymus or spleen was used, while tail DNA of the respective mouse was used as a reference germline material. Tumor-specific somatic mutations were determined by *mutect* and *varscan* analysis. The color code indicates the type of alteration and the genotype of the leukemic mouse in each case. SPF, specific pathogen-free.

Our findings demonstrated that ETV6-RUNX1-associated B-ALL does not originate in the committed B-cell compartment. Therefore, we tested the transforming potential of *ETV6-RUNX1* expression in earlier hematopoietic stem or progenitor cells (HS/PCs). To this end, we used *ETV6-^*ETV*6–RUNX1^* mice crossed with *Sca1-Cre* mice (Mainardi et al., 2014) to initiate *ETV6-RUNX1* expression in hematopoietic stem cells (HSCs) in which the *Sca1* (*Ly6a*; NCBI Gene ID: 110454) promoter is active and to maintain the expression in all descending hematopoietic cells (Figure 1B). *ETV6-RUNX1* expression resulted in striking alterations restricted to the B-cell compartment. A significant and specific decrease of pro/preB-cells (B220^*low*^ IgM^–^) and recirculating B-cells (B220^++^IgM^+^) was evident in *ETV6-^*ETV*6–RUNX1^* + *Sca1-Cre* mice compared to age-matched WT littermates (Figure 2B). The B-cells in peripheral blood were also significantly reduced (Figure 2C). *ETV6-^*ETV*6–RUNX1^* + *Sca1-Cre* mice had a shorter lifespan when exposed to natural infections than their WT littermates [Figure 2A; *p* < 0.0001; log-rank (Mantel–Cox) test; Supplementary Table 1] due to the development of specific lymphoid leukemias, including both T- (34.4%; 11/32) between 6.2 and 14.2 months of age and B-ALL (6.3%; 2/32) between 19.5 and 20.1 months of age. Notably, *ETV6-^*ETV*6–RUNX1^* + *Sca1-Cre* mice did not develop any myeloid malignancies. T-ALL manifested as thymoma, splenomegaly, and disrupted thymic, liver, kidney, small intestine, and splenic architectures (Figures 3A,B and Supplementary Figure 4). FACS analysis of leukemic cells revealed an immature CD8^+^CD4^±^ cell surface phenotype (Figure 3C), with clonal immature T-cell receptor (TCR) rearrangement (Supplementary Figure 5). T-ALL development in *ETV6-^*ETV*6–RUNX1^* + *Sca1-Cre* mice is a characteristic of the mouse model as ETV6-RUNX1+ T-ALL never occurs in humans. B-ALL manifested as disruption of splenic architecture due to blast infiltration and appearance of blast cells in the lung (Supplementary Figure 6). *ETV6-^*ETV*6–RUNX1^* + *Sca1-Cre* B-ALLs displayed clonal immature BCR rearrangement (Supplementary Figure 7). FACS analysis revealed a CD19^+^B220^+^IgM^+^ cell surface phenotype for tumor cells in BM and PB (Figure 3D). Since *ETV6-RUNX1* expression was maintained constitutively in all hematopoietic cells in the *ETV6-^*ETV*6–RUNX1^* + *Sca1-Cre* model (Figure 1B), the results suggested that *ETV6-RUNX1* restricted the tumor type to lymphoid ALL, and a second hit further determined the ALL subtype. When we analyzed the mutational landscape of the generated ALLs by whole-exome sequencing of *ETV6-^*ETV*6–RUNX1^* + *Sca1-Cre* T-ALL (*n* = 10) and *ETV6-^*ETV*6–RUNX1^* + *Sca1-Cre* B-ALL (*n* = 2) (Figure 4), the mutations detected in T-ALL showed a significant overlap with human T-ALL (Liu et al., 2017), with *Notch1* (HGNC:7881) and *Bcl11b* (HGNC:13222) mutations identified recurrently (Figure 4). By contrast, T-ALL-specific mutations were absent in B-ALL.

### *ETV6-RUNX1* B-ALL Is Not Triggered by the Second Hit at the Committed B-Cell Stage

**FIGURE 5:**
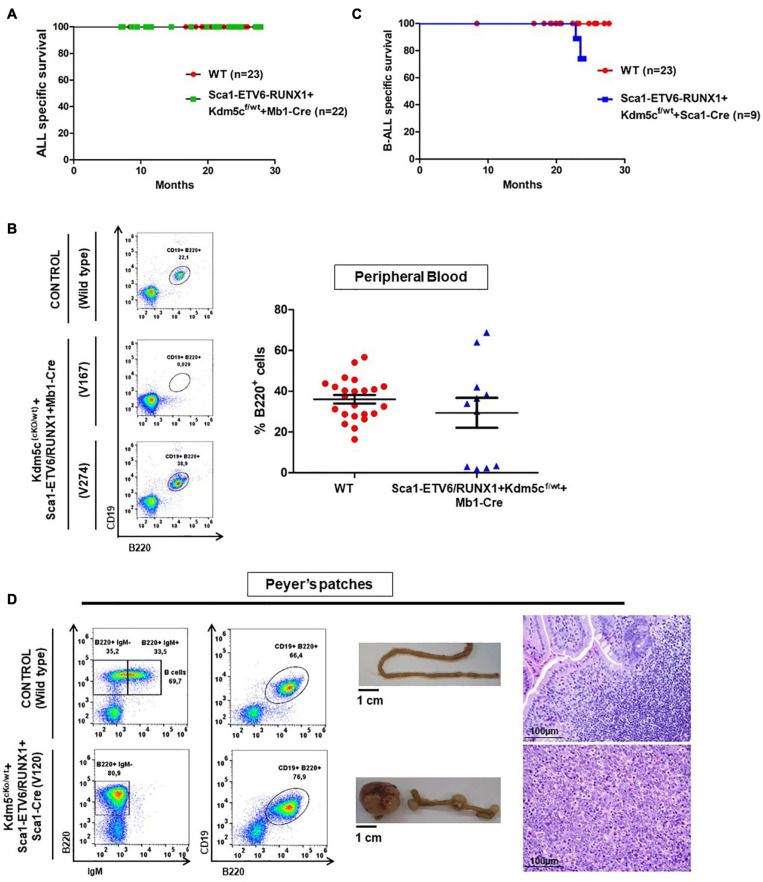
*Kdm5c* loss-of-function in hematopoietic stem cells contributes to the clonal evolution of an ETV6-RUNX1 preleukemic clone to B-ALL without the need of infection exposure. **(A)** Leukemia-specific survival of Sca1-ETV6/RUNX1 + Kdm5c^f/wt^ + Mb1-Cre (*n* = 22) compared to control littermate WT mice (red points, *n* = 23). **(B)** Peripheral blood fluorescence-activated cell sorting analysis in Sca1-ETV6/RUNX1 + Kdm5c^*f/wt*^ + Mb1-Cre (*n* = 11) compared to age-matched (6 months old) control wild-type (WT, *n* = 23) mice. The B-cell defect does not have a complete penetrance in Sca1-ETV6/RUNX1 + Kdm5c^*f/wt*^ + Mb1-Cre mice. **(C)** Leukemia-specific survival of Sca1-*ETV6-RUNX1* + Kdm5c^*f/wt*^ + Sca1-Cre (blue line, *n* = 9), showing a not significantly (log-rank test *p*-value = 0.1695) shortened lifespan compared to control littermate WT mice (red line, *n* = 23) as a result of B-ALL development. **(D)** Flow cytometric analysis of B-cell subsets in the Peyer’s patches of diseased Sca1-*ETV6-RUNX1* + Kdm5c^*f/wt*^ + Sca1-Cre mice with B-ALL. Representative plots of cell subsets are shown. These exhibited the accumulation of CD19^+^B220^+^IgM^–^ -tumoral B-cells. The flow cytometric images are representative of nine mice analyzed. The accumulation of tumoral B-cells can be observed macroscopically (image of the small intestine is shown) and microscopically (hematoxylin and eosin staining of the infiltrated tissue). WT mouse is shown for reference in each case. Images are photographed at ×400 magnification.

We next addressed whether the transformation of *ETV6-RUNX1* preleukemic cells can be triggered by the introduction of a second hit in committed B-cells. To this end, *Kdm5c* loss, previously identified to be missense mutated in the murine *ETV6-RUNX1*+ B-ALL and in human relapse ETV6-RUNX1+ B-ALL (Rodriguez-Hernandez et al., 2017a), was introduced in the B-cell compartment of Sca1*-ETV6-RUNX1* mice by crossing with a targeted Kdm5c^*f/wt*^ mouse line obtained from a public repository (EMMA). This allowed a Cre-dependent *Kdm5c* deletion of exons 15–17 in the precursor B-cell lineage by crossing with an *Mb1-Cre* mouse strain. We monitored the cohorts of Sca1-*ETV6-RUNX1* + Kdm5c^*f/wt*^ + Mb1-Cre and control WT mice born and kept in the SPF environment throughout their lifespans (*n* = 22; followed up to 2 years) (Figures 1E, 5A and Supplementary Table 1). None of the Sca1-*ETV6-RUNX1* + Kdm5c^*f/wt*^ + Mb1-Cre mice developed B-ALL. However, *Kdm5c* loss resulted in B-cell-specific toxicity because, in a significant proportion of Sca1-*ETV6-RUNX1* + Kdm5c^*f/wt*^ + Mb1-Cre mice (4/11; 36.3%), B-cells were lacking (Figure 5B and Supplementary Figure 8). The lack of B-ALL in Sca1-*ETV6-RUNX1* + Kdm5c^*f/wt*^ + Mb1-Cre mice suggests that *Kdm5c* loss-of-function at the B-cell stage does not contribute to the malignant transformation of an *ETV6-RUNX1*+ preleukemic clone.

### *ETV6-RUNX1* B-ALL Is Triggered by the Second Hit at the HS/PC Stage

To test if B-ALL can be specifically triggered by a second hit at the stem cell or early progenitor stage, we introduced *Kdm5c* loss into Sca1*-ETV6-RUNX1* mice by crossing with Kdm5c^*f/wt*^ mice (Figure 1D). *Kdm5c* loss-of-function in HSCs, in contrast to B-cells, did not adversely affect B-cell development (Supplementary Figure 9).

B-ALL development (22%; two out of nine) was observed in the generated Sca1-*ETV6-RUNX1* + Kdm5c^*f/wt*^ + Sca1-Cre mice born and kept in the SPF environment (*n* = 9; observed for up to 2 years) (Figure 5C and Supplementary Table 1). By contrast, Sca1-*ETV6-RUNX1* mice never develop B-ALL under SPF conditions (Rodriguez-Hernandez et al., 2017a). Due to the low incidence of the B-ALL disease, the overall survival of Sca1-*ETV6-RUNX1* + Kdm5c^*f/wt*^ + Sca1-Cre was not significantly reduced compared to WT mice [*p*-value = 0.1695; log-rank (Mantel–Cox) test Figure 5C]. The leukemia onset in *Sca1-ETV6-RUNX1* + *Kdm5cf^/wt^* + *Sca1-Cre* mice housed in SPF conditions was similar to the one arising in *Sca1-ETV6-RUNX1* mice exposed to infection (Rodriguez-Hernandez et al., 2017a), indicating that loss of *Kdm5c* may be involved in the leukemogenesis of *ETV6-RUNX1*+ ALL but is not the main second hit critical for disease development. Sca1-*ETV6-RUNX1* + Kdm5c^*f/wt*^ + Sca1-Cre B-ALLs displayed clonal immature BCR rearrangement (Supplementary Figure 10), and FACS analysis revealed a CD19^+^B220^+^IgM^–^ cell surface phenotype of the tumor cells in BM, PB, and spleen (Supplementary Figure 11) with the capability to infiltrate other tissues like the spleen, liver, and small intestine (Figure 5D and Supplementary Figure 12).

### The Second Hit Determines the Cell Fate of *ETV6-RUNX1-*Positive Leukemia

**FIGURE 6:**
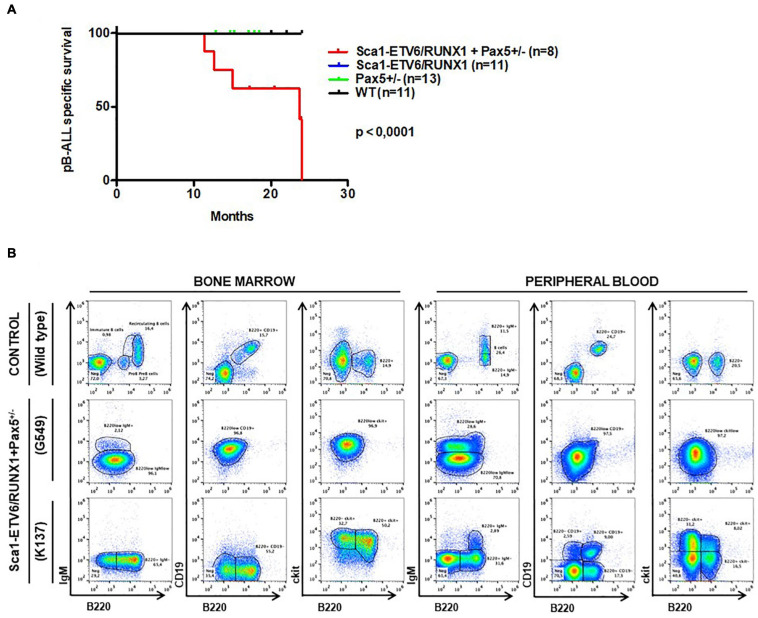
*Pax5-loss* triggers ETV6-RUNX1 preleukemic transformation without need of infection exposure. **(A)** Leukemia-specific survival of Sca1-*ETV6-RUNX1* + Pax5-het mice (red line, *n* = 8), showing a significantly (log-rank test *p*-value = 0.0001) shortened lifespan as a result of B-ALL development compared to control littermate WT mice (black line, *n* = 11), Pax5-het mice (green line, *n* = 13), and Sca1-*ETV6-RUNX1* mice (blue line, *n* = 11). **(B)** Flow cytometric analysis of B-cell subsets in the bone marrow and peripheral blood of diseased Sca1-*ETV6-RUNX1* + Pax5-het mice with B-ALL. Representative plots of cell subsets are shown. These exhibited the accumulation of CD19^±^B220^+^cKit^±^IgM^–^ tumoral B-cells.

Whole-exome sequencing of Sca1-*ETV6-RUNX1* + *Kdm5c^*f/wt*^* + *Sca1-Cre* B-ALL (*n* = 2; Figure 4) showed an overlap with genes or pathways mutated in B-ALL and an absence of T-ALL-specific mutations, such as *Notch1*. Our data, therefore, indicated that second molecular alterations may confer cell identity to *ETV6-RUNX1*+ leukemia. To corroborate this hypothesis, we tested whether the heterozygous loss of *Pax5* (*Pax5-het*), a common second hit identified in murine and human B-ALL-associated *ETV6-RUNX1* (Heltemes-Harris et al., 2011; Papaemmanuil et al., 2014; Martin-Lorenzo et al., 2018), would restrict the tumor cell type to B-ALL in Sca1-*ETV6-RUNX1* mice, similarly to *Kdm5c* loss. We generated Sca1-*ETV6-RUNX1* + Pax5-het mice in an SPF environment to avoid the induction of second hits due to the exposure to infections (Figure 1C). Double Sca1-ETV6-RUNX1 + Pax5-het mice developed B-ALL (62.5%; five out of eight); as a result, they had shorter lifespans than their WT, Pax5-het, and Sca1-*ETV6-RUNX1* littermates [Figure 6A; *p* < 0.0001; log-rank (Mantel–Cox) test] when kept in an SPF environment. Pax5-het mice alone or Sca1-ETV6-RUNX1 mice never develop B-ALL in an SPF facility (Martin-Lorenzo et al., 2015; Rodriguez-Hernandez et al., 2017a). Thus, the combination of the first hit “Sca1-ETV6-RUNX1” and the second hit “Pax5-het” is what leads to B-ALL development without the need of exposure to infections as the second hit is already present. FACS analysis revealed a CD19^+^B220^+^IgM^–^ cell surface phenotype of the tumor cells. They extended into the BM, PB, and spleen, infiltrated other tissues, and displayed clonal immature BCR rearrangements (Figure 6B, Supplementary Figure 13, and Supplementary Figure 14). Whole-exome sequencing of Sca1-*ETV6-RUNX1* + *Pax5-het* B-ALL (Figure 4) showed mutations affecting the Pax5 gene.

**FIGURE 7:**
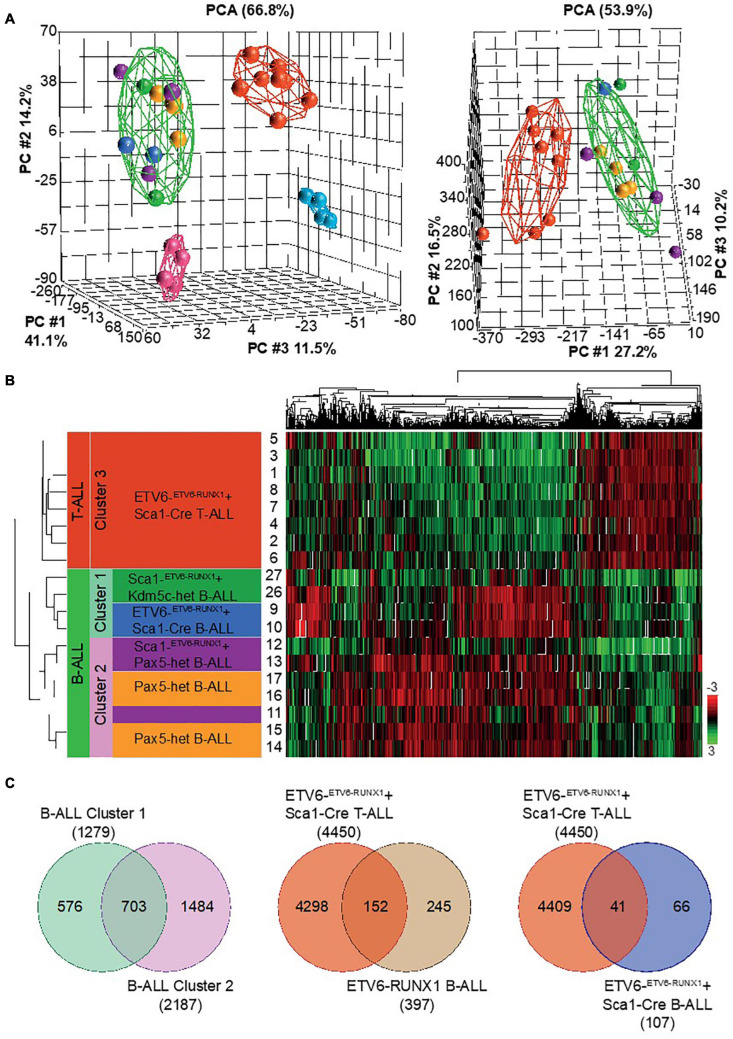
RNA sequencing (RNA-Seq) to globally quantify the similarity between Kdm5c-driven and Pax5-driven B-ALL-associated ETV6-RUNX1. We examined leukemic cell populations from B-ALL (*n* = 2) and T-ALL (*n* = 8) leukemias from ETV6-^*ETV*6– *RUNX*1^ + Sca1-Cre mice, B-ALL (*n* = 2) from Sca1-ETV6-RUNX1 + Kdm5c^*f/wt*^ + Sca1-Cre mice (*n* = 2), B-ALL (*n* = 3) from Sca1-ETV6-RUNX1 + Pax5-het mice, and B-ALL (*n* = 4) from Pax5-het mice. We used wild-type (WT) FACS-sorted pro-B-cells (*n* = 4) and thymus T-cells (*n* = 4) as controls. **(A)** Principal component analysis of mouse leukemias with (left) and without (right) controls. The spheres are colored based on genetic background: ETV6-^*ETV*6–RUNX1^ + Sca1-Cre T-ALL 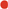, ETV6-^*ETV*6–RUNX1^ + Sca1-Cre B-ALL 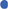, Pax5-het B-ALL 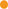, Sca1-^*ETV*6–RUNX1^ + Kdm5c-het B-ALL 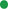, Sca1-^*ETV*6–RUNX1^ + Pax5-het B-ALL 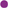, WT T cells 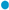, and WT pro-B-cells 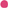. Broad disease terms were used to generate elipses around T-ALL 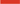, B-ALL 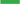, WT T cells 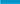, and WT pro-B-cells 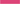. **(B)** Unsupervised hierarchical clustering of top 10% genes based on highest standard deviation (*n* = 1,314) and clustered using Pearson’s dissimilarity algorithm. The samples were divided into three clusters based on dendrogram distances. **(C)** Venn diagrams showing genes in common. *T*-tests were performed by comparing grouped ALL tumors against an appropriate control. Significance was determined based on a fold change ± 2 and a false discovery rate *q*-value ≤ 0.05.

If the nature of the second hit confers tumor cell identity to *ETV6-RUNX1*+ leukemia, we hypothesized that *ETV6-RUNX1*+ B-ALLs triggered by the loss of either Pax5 or Kdm5c should differ from one another. To analyze this, we used RNA sequencing (RNA-Seq) and compared the gene expression patterns. We examined leukemic cell populations from B-ALL (*n* = 2) and T-ALL (*n* = 8) from ETV6-^*ETV*6–RUNX1^ + Sca1-Cre mice, B-ALL (*n* = 2) from Sca1-ETV6-RUNX1 + Kdm5c^*f/wt*^ + Sca1-Cre mice (*n* = 2), B-ALL (*n* = 3) from Sca1-ETV6-RUNX1 + Pax5-het mice, and B-ALL (*n* = 4) from Pax5-het mice (Martin-Lorenzo et al., 2015). We used WT FACS-sorted pro-B-cells (*n* = 4) and thymus T-cells (*n* = 4) as controls. The data were analyzed by principal component analysis (PCA) as a measure of the overall similarity between samples (Figure 7A). As expected, unsupervised PCA clearly separated ETV6-RUNX1+ B-ALL from T-ALL into distinct clusters (Figure 7A). Remarkably, B-ALLs were also clearly separable based on the second hit (Figures 7A–C and Supplementary Table 2). Sca1-ETV6-RUNX1 + Pax5-het B-ALL clustered together with B-ALL that originated as a result of *Pax5* loss (Martin-Lorenzo et al., 2015), whereas Sca1-ETV6-RUNX1 + Kdm5c^*f/wt*^ + Sca1-Cre B-ALL clustered with ETV6-^*ETV*6–RUNX1^ + Sca1-Cre B-ALL. Collectively, our data indicate that ETV6-RUNX1+ ALL originates from preleukemic hematopoietic precursor cells and that the second hit further determines cell identity and tumor subtype.

## Discussion

*ETV6-RUNX1* is associated with the most common subtype of childhood leukemia. However, the incidence of the *ETV6-RUNX1* fusion is about 100-fold higher than the incidence of *ETV6-RUNX1*+ B-ALL in children (Mori et al., 2002; Schafer et al., 2018), and a specific environmental context seems to be necessary to turn preleukemic cells into overt leukemia (Greaves, 2018; Cobaleda et al., 2021a,b). Indeed we have recently presented *in vivo* genetic evidence showing the clonal evolution of an *ETV6-RUNX1* preleukemic clone to an irreversibly transformed state after a natural infection exposure (Rodriguez-Hernandez et al., 2017a). These and other findings suggest that B-ALL in genetic carriers might be a preventable disease (Martin-Lorenzo et al., 2015; Rodriguez-Hernandez et al., 2017a,b, 2019; Cobaleda et al., 2021a,b). To develop methods for the potential prevention of the disease onset, it would be necessary to first understand the sequential events leading to B-ALL. However, the initial steps of the disease usually pass unnoticed in children, and at the time of diagnosis, the deconvolution of the timing of sequential events leading to B-ALL is hampered by the presence of a wide range of accumulated oncogenic driver and bystander mutations. Therefore, we used genetically engineered mice to specifically address this question and to model the early steps of the disease.

Currently, a two-hit model of *ETV6-RUNX1* leukemogenesis is assumed, namely: (I) ETV6-RUNX1 creates a preleukemic cell pool and (II) secondary events cooperate and transform committed pre-B-cells (Greaves, 2018; Cobaleda et al., 2021a,b). A preleukemic cell pool was created when the *ETV6-RUNX1* fusion gene was introduced into human cord blood cells (Hong et al., 2008) or murine HSCs (Schindler et al., 2009), but the fusion gene alone was not sufficient to cause leukemic transformation. A recent work shows that a preleukemic cell pool can also be generated by introducing *ETV6-RUNX1* into a developmentally restricted B-cell progenitor unique to early embryonic life (Boiers et al., 2018). The preleukemic oncogenic lesion is stably maintained as a single alteration in an abnormal cell population and will only cause leukemia when a cooperating second hit occurs.

Extending previous studies, we provide clear evidence that *ETV6-RUNX1* fails to induce B-ALL when expressed in committed B-cells regardless of a cooperating second hit (natural infection). Under the same conditions, *ETV6-RUNX1* expressed in hematopoietic progenitors readily induces leukemia if the mice are kept in a natural infection environment or if a second mutation (*Kdm5c* or *Pax5* loss) occurs in close succession. Transformation fails when this second hit occurs at a later stage. This was demonstrated by a targeted loss of *Kdm5c* in committed B-cells. Taken together, *ETV6-RUNX1* expression and a second genetic or environmental hit must occur at the hematopoietic progenitor stage for leukemic transformation to take place.

Our data further demonstrate that the presence of ETV6-RUNX1 is necessary for the early stages of transformation but that the final tumor phenotype is determined by the second hit experienced by the hematopoietic/precursor experiences. Accordingly, ETV6-RUNX1-expressing hematopoietic progenitors gave rise to both T- and B-cell ALL in the presence of a natural infection environment. These T-ALLs presented with a mutational landscape similar to human T-ALL, including *Notch1* mutations. Although ETV6-RUNX1 is always associated to B-ALL development in humans, the preleukemic cell of origin in children seems to have T-cell potential. Common ancestral clones containing partial TCR rearrangements have been identified through single-cell sequencing of ETV6-RUNX1 ALL in monochorionic twins (Alpar et al., 2015). Why this type of childhood ALL is restricted to B-ALL is still unclear. Remarkably, *Notch1* mutations that direct toward a T-ALL fate are not observed in human ETV6-RUNX1+ leukemias (Papaemmanuil et al., 2014).

In our models, a B-cell tumor fate could be determined by the targeted loss of either *Kdm5c* or *Pax5*, even without additional environmental infection exposure, but these two secondary mutation events do not have the same relevance in the development of B-ALL according to the result presented in this study. Loss of *Kdm5c* may be involved in the leukemogenesis of ETV6/RUNX1+ ALL, but it is doubtful whether it is a main second hit driving leukemia, as in humans it has been only observed in a relapse sample (Rodriguez-Hernandez et al., 2017a). Additionally, the onset of the disease in the *Sca1-ETV6-RUNX1* + *Kdm5cf^/wt^* + *Sca1-Cre* mouse model (22%) should be earlier and more frequent than in the mouse that only has the first hit (*Sca1-ETV6-RUNX1 mice*) and, due to exposure to infections, acquires the second hit (10.75%) (Rodriguez-Hernandez et al., 2017a). On the contrary, the loss of *Pax5* is critical for ETV6/RUNX1+ B-ALL developed, as the onset of the disease in *Sca1-ETV6-RUNX1* + *Pax5-het* mice (62.5%) is drastically increased even in the absence of exposure to infections, compared to *Sca1-ETV6-RUNX1* mice (10.75%) and exposed to infections (Rodriguez-Hernandez et al., 2017a).

That these second hits determined clearly separable B-cell fates was indicated by a distinct cluster formation in RNA expression analysis. RNA sequencing further demonstrated that B-ALLs caused by loss of *Pax5* grouped closely together (*Sca1-ETV6-RUNX1* + Pax5-het mice and *Pax5-het* mice under infection exposure). These findings suggest that, in the presence of Pax5 loss, both *ETV6-RUNX1* and natural infection exposure trigger phenotypically similar B-ALLs. Taken together, our data demonstrate that ETV6-RUNX1 promotes tumorigenesis in a manner distinct from other more dominant oncogenes. ETV6-RUNX1 generates a pool of susceptible preleukemic cells with lymphoid developmental potential. The final disease phenotype is determined by the specific secondary hit. The rareness of the second hit at the specifically vulnerable progenitor state may explain the low penetrance of B-ALL in ETV6-RUNX1+ genetic carriers. Our findings have important implications for the understanding and potential therapeutic targeting of the preleukemic state in children.

## Data Availability Statement

The datasets presented in this study can be found in online repositories. The names of the repository/repositories and accession number(s) are as follows: Gene Expression Omnibus GSE141112.

## Ethics Statement

The animal study was reviewed and approved by Servicio de Trazabilidad e Higiene Ganadera de la Dirección General de Producción Agropecuaria e Infraestructuras Agrarias - Junta de Castilla y León - (Ref: 000186).

## Author Contributions

CV-D and IS-G designed the initial conception of the project. GR-H, AC-G, MI-H, DP, JR-G, SA-A, AO, OB, PP-M, SR, MG, and FG contributed to the development of the methodology. OB, MG, FG, and CV-D performed the pathology review. GR-H, DP, HH, TE, IS-G, and CV-D contributed to the analysis and interpretation of the data (e.g., statistical analysis, biostatistics, and computational analysis). GR-H, AC-G, MI-H, DP, JR-G, SA-A, AO, OB, PP-M, SR, MG, FG, HH, TE, IS-G, and CV-D prepared the manuscript. GR-H, IS-G, and CV-D contributed to the administrative, technical, or material support (i.e., reporting or organizing data and constructing databases). IS-G and CV-D supervised the study. All authors contributed to the article and approved the submitted version.

## Conflict of Interest

The authors declare that the research was conducted in the absence of any commercial or financial relationships that could be construed as a potential conflict of interest.

## Footnotes

1 http://broadinstittute.github.io/picard

2 https://pecan.stjude.cloud/

3 https://www.ncbi.nlm.nih.gov/geo/query/acc.cgi?acc=GSE141112
