# Supplementary material for: The Second Oncogenic Hit Determines the Cell Fate of ETV6-RUNX1 Positive Leukemia
Source: Front Cell Dev Biol. 2021 Jul 15;9:704591. doi: 10.3389/fcell.2021.704591 (PMC8320889; doi:10.3389/fcell.2021.704591)
Supplement: Supplementary file 2 [file Data_Sheet_1.pdf]

Supplementary Material

Supplementary Data:

Rodríguez-Hernández et al. The second oncogenic hit determines the cell fate of ETV6-RUNX1 positive leukemia

Supplementary Tables:

Supplementary Table 1:

GENOTYPE	TOTAL	ALL	T-ALL	B-ALL	FACILITY
**ETV6-$^{ETV6\text{-}RUNX1}$ +Mb1-Cre**	31	0	0	0	CF
**ETV6-$^{ETV6\text{-}RUNX1}$ +Sca1-Cre**	32	13 (40.63%)	11 (34.38%)	2 (6.25%)	CF
**Sca1-ETV6-RUNX1 + Kdm5c$^{f/wt}$+Mb1-Cre**	22	0	0	0	SPF
**Sca1-ETV6-RUNX1 + Kdm5c$^{f/wt}$+Sca1-Cre**	9	2 (22.2%)	0	2 (22.2%)	SPF
**Sca1-ETV6-RUNX1 + Pax5-het**	8	5 (62.50%)	0	5 (62.50%)	SPF

CF: Conventional Facility; SPF: Specific Pathogen Free

Supplementary Table 1: Incidence of disease mice in ETV6-$^{ETV6\text{-}RUNX1}$+Sca1-Cre; ETV6-$^{ETV6\text{-}RUNX1}$+Mb1-Cre mice, Kdm5c $^{f/wt}$ + Sca1-ETV6/RUNX1+ Mb1-Cre mice and Sca1-*ETV6/RUNX1*+ Kdm5c$^{f/wt}$ + Sca1-Cre mice.

Supplementary Table 2: List of all genes from comparisons in Figure 7C.

Supplementary Table 2 is presented as an independent Excel file.

Supplementary Table 3: List of resources used in the study.

REAGENT or RESOURCE		SOURCE
**Antibodies**		
CD4 (RM4-5)	Flow cytometry	Biolegend
CD8a (53-6.7)	Flow cytometry	Biolegend
CD3Ɛ (145-2C11)	Flow cytometry	Biolegend
B220 (RA3-6B2)	Flow cytometry	Biolegend
IgM (RMM-1)	Flow cytometry	Biolegend
CD19 (1D3)	Flow cytometry	Biolegend
CD11b/Mac1 (M1/70)	Flow cytometry	Biolegend
Ly-6G/Gr1 (RB6-8C5)	Flow cytometry	Biolegend
CD25 (PC61)	Flow cytometry	Biolegend
CD117/c-Kit (2B8)	Flow cytometry	Biolegend
CD16/CD32 (2.4G2)	Flow cytometry	BD Biosciences
RUNX1	Western Blot	Abcam, ab23980
β-Actin	Western Blot	C4: sc-47778
**Experimental Models: Organisms/Strains**		
Mice: ETV6-$^{ETV6\text{-}RUNX1}$ (C57BL6J/CBA)	Schindler et al., 2009	Schindler et al., 2009
Mice: Mb1-Cre (C57BL6J/CBA)	Hobeika et al., 2006	Hobeika et al., 2006
Mice: Sca1-Cre (C57BL6J/CBA)	Mainardi et al., 2014	Mainardi et al., 2014
Mice: Kdm5c$^{f/wt}$ (C57BL/6N)	Kdm5c<tm1c(EUCOMM)Hmgu>/Ics	EMMA public repository (Strain ID: EMMA:06928)
Mice: Sca1-ETV6-RUNX1 (C57BL6J/CBA)	Rodriguez-Hernandez et al., 2017a	Rodriguez-Hernandez et al., 2017a
Mice: Pax5-het (C57BL6J/CBA)	Urbanek P et al., 1994	Urbanek P et al., 1994
**Experimental Models: Cell Lines**		
REH cells	ATCC CRL-8286	ETV6-RUNX1 translocation positive B lymphoblastic cell line
**Oligonucleotides**		
V_HJ558	forward	CGAGCTCTCCARCACAGCCTWCATGCARCTCARC
	reverse	GTCTAGATTCTCACAAGAGTCCGATAGACCCTGG
V_H7183	forward	CGGTACCAAGAASAMCCTGTWCCTGCAAATGASC
	reverse	GTCTAGATTCTCACAAGAGTCCGATAGACCCTGG
V_HQ52	forward	CGGTACCAGACTGARCATCASCAAGGACAAYTCC
	reverse	GTCTAGATTCTCACAAGAGTCCGATAGACCCTGG
DH	forward	TTCAAAGCACAATGCCTGGCT
	reverse	GTCTAGATTCTCACAAGAGTCCGATAGACCCTGG
Cμ	forward	TGGCCATGGGCTGCCTAGCCCGGGACTT
	reverse	GCCTGACTGAGCTCACACAAGGAGGA
**Software and Algorithms**		
FlowJo (version 10.1.r7)	FlowJo, LLC	https://www.flowjo.com/solutions/flowjo
GraphPad Prism (version 6.0)	GraphPad Software Inc	https://www.graphpad.com/scientific-software/prism/
Partek Flow	Partek Incorporated, Missouri, USA	https://www.partek.com/
Bowtie 2 v2.2.5 aligner	Johns Hopkins University	http://bowtie-bio.sourceforge.net/bowtie2/index.shtml
**Data collection**		
BD AccuriTM C6 Flow Cytometer	https://www.bdbiosciences.com/en-us/products/instruments/flow-cytometers/research-cell-analyzers/bd-accuri-c6-plus	
2100 Bioanalyzer system	https://www.agilent.com/en/product/automated-electrophoresis/bioanalyzer-systems/bioanalyzer-instrument	
HiSeq 2500 (Illumina)	https://emea.illumina.com/systems/sequencing-platforms/hiseq-2500.html	
**Deposited Data**		
RNA sequencing data	GEO Series accession number: GSE141112	https://www.ncbi.nlm.nih.gov/geo/query/acc.cgi
**Online available Data**		
St. Jude Cloud PeCan	human B- or T-ALL	https://pecan.stjude.cloud/

Supplementary Figures:

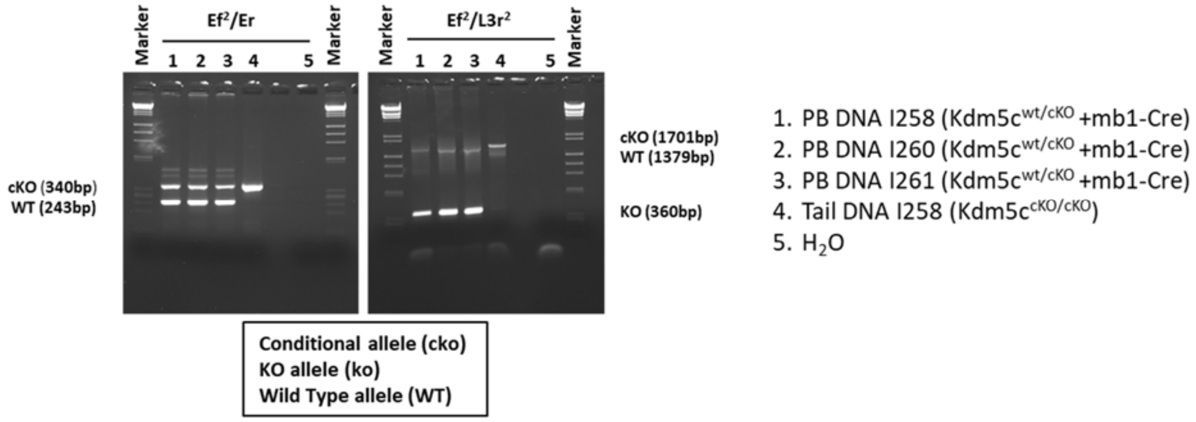

Supplementary Figure 1. PCR to detect deletion of Kdm5c mediated by Mb1-cre in peripheral blood of kdm5c$^{wt/cKO}$ +Mb1-Cre mice. PCR analysis confirms deletion of the kdm5c allele in peripheral blood of kdm5$^{cko/wt}$ mice when crossed with Mb1-Cre mice. As expected, there are still some cells in the peripheral blood that have not deleted the conditional kdm5c allele (cKO band).

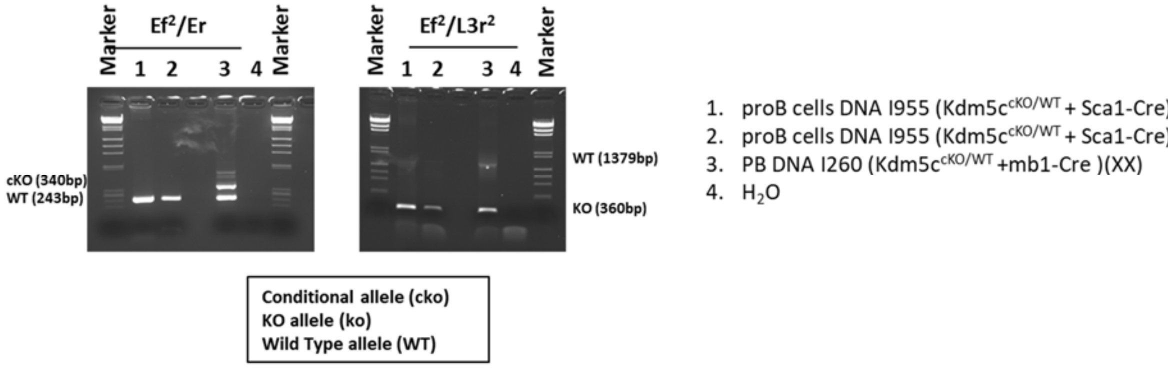

Supplementary Figure 2. PCR to detect deletion of Kdm5c mediated by Sca1-Cre in pro-B cells of kdm5c$^{wt/cKO}$ +Sca1-Cre mice. PCR analysis confirms deletion of the kdm5c allele in bone marrow proB cells of kdm5$^{cko/wt}$ mice when crossed with Sca1-Cre mice.

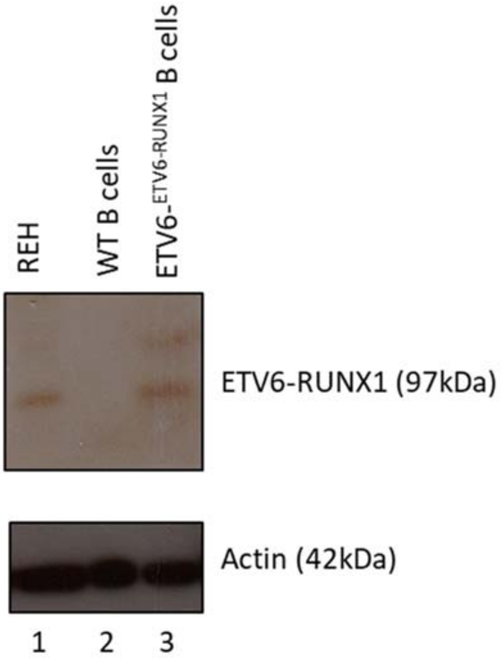

Supplementary Figure 3. Western blot of ETV6-RUNX1 protein from FACS sorted B cells derived from ETV6-$^{ETV6\text{-}RUNX1}$+Mb1-Cre mice. Lane 1, positive control: human B-ALL cell line carrying ETV6-RUNX1 fusion (REH); lane 2, negative control: FACS sorted B cells derived from WT mice; lane 3, FACS sorted B cells derived from ETV6-$^{ETV6\text{-}RUNX1}$+Mb1-Cre mice.

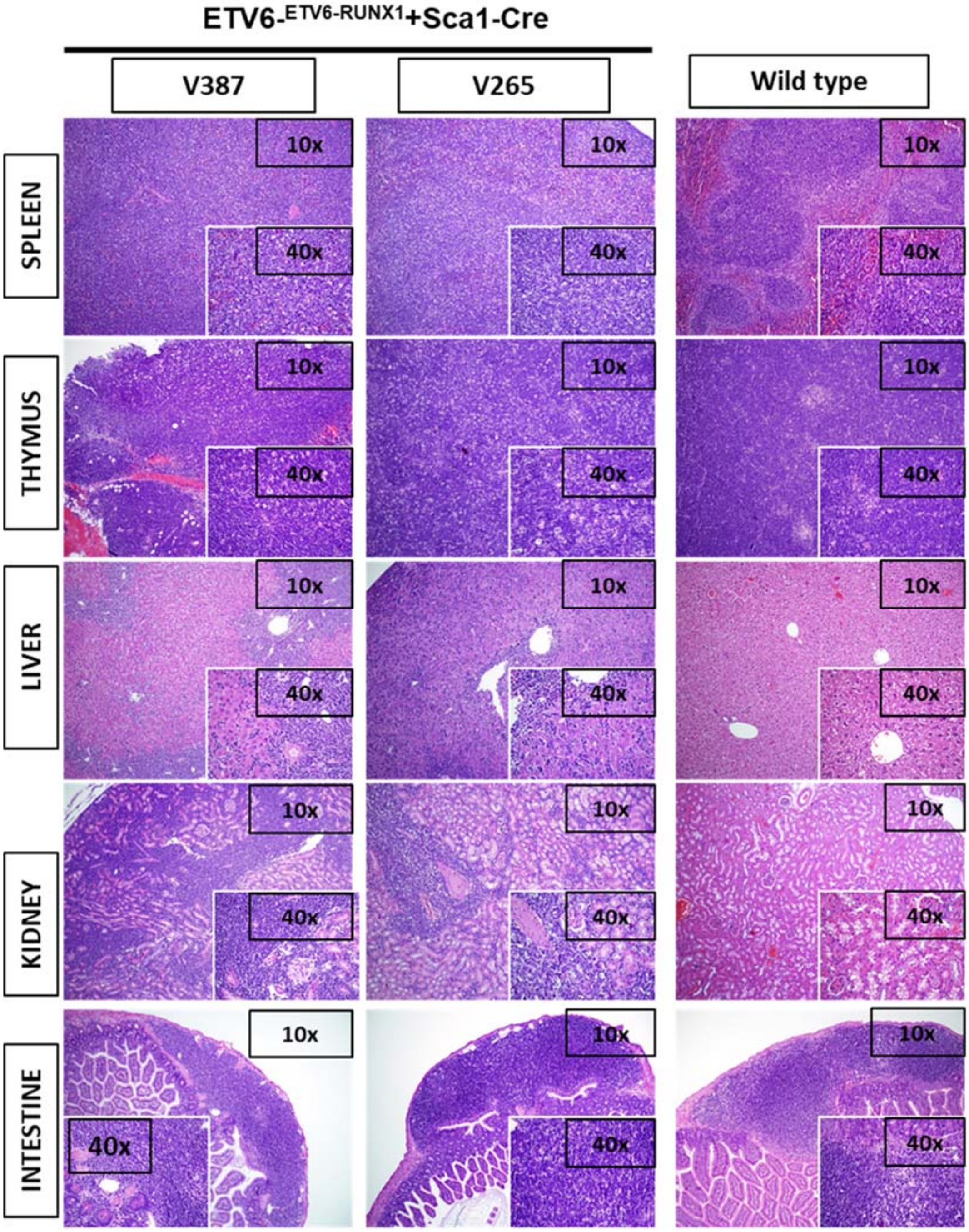

Supplementary Figure 4. Histological images of ETV6-$^{ETV6\text{-}RUNX1}$+Sca1-Cre disease mice with T-ALL. Hematoxylin and eosin staining of spleen, thymus, liver, kidney and small intestine from wild-type and tumor-bearing ETV6-$^{ETV6\text{-}RUNX1}$+Sca1-Cre mice shows loss of normal architecture resulting from effacement with cells morphologically resembling lymphoblasts.

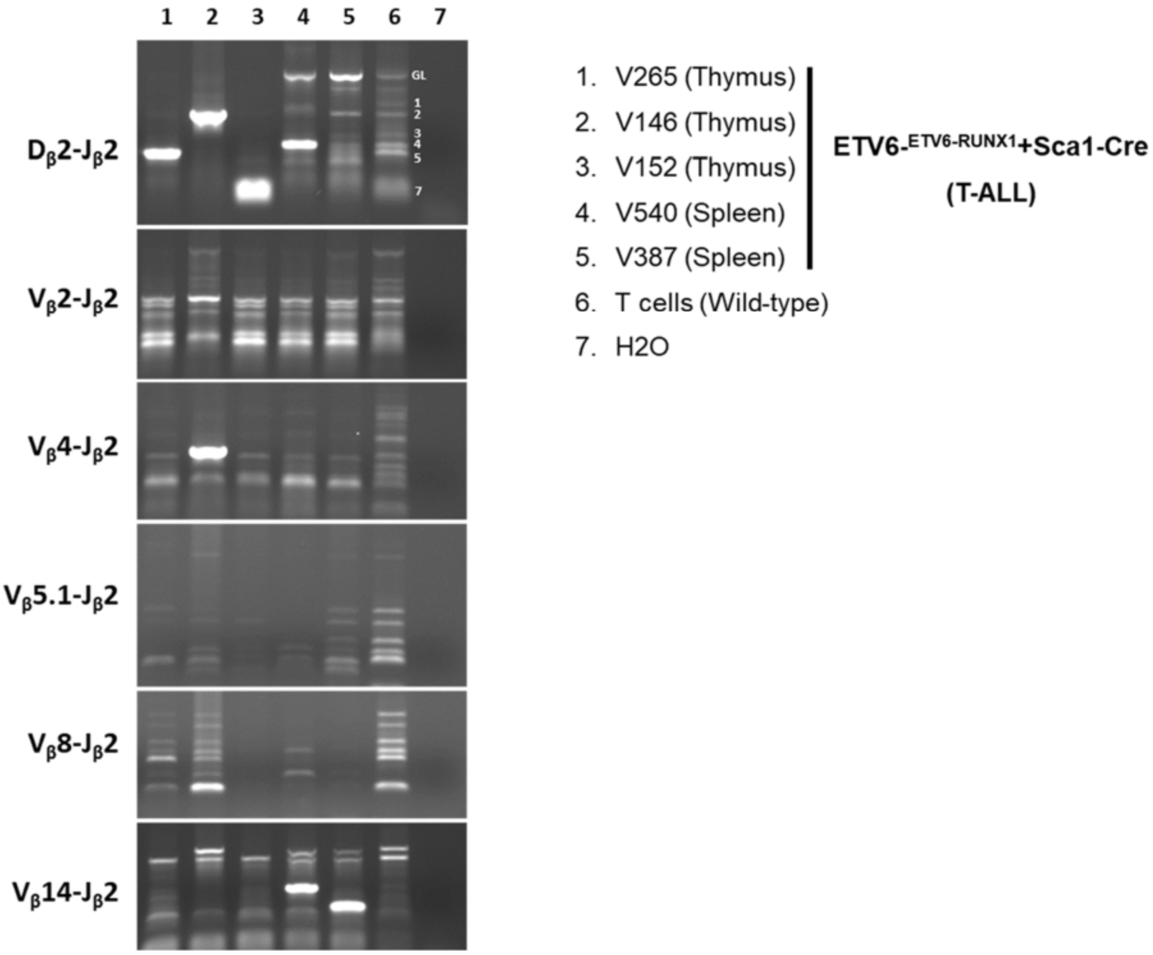

Supplementary Figure 5. Mouse TCR Rearrangement analyzed by PCR of ETV6-$^{\text{ETV6-RUNX1}}$+Sca1-Cre disease mice with T-ALL. TCR clonality in ETV6-$^{\text{ETV6-RUNX1}}$+Sca1-Cre mice. PCR analysis of *TCR* gene rearrangements in infiltrated thymuses of diseased ETV6-$^{\text{ETV6-RUNX1}}$+Sca1-Cre leukemic mice. Sorted DP T cells from the thymus of healthy mice served as a control for polyclonal TCR rearrangements. Leukemic thymus shows an increased clonality within their TCR repertoire (indicated by the code number of each mouse analyzed).

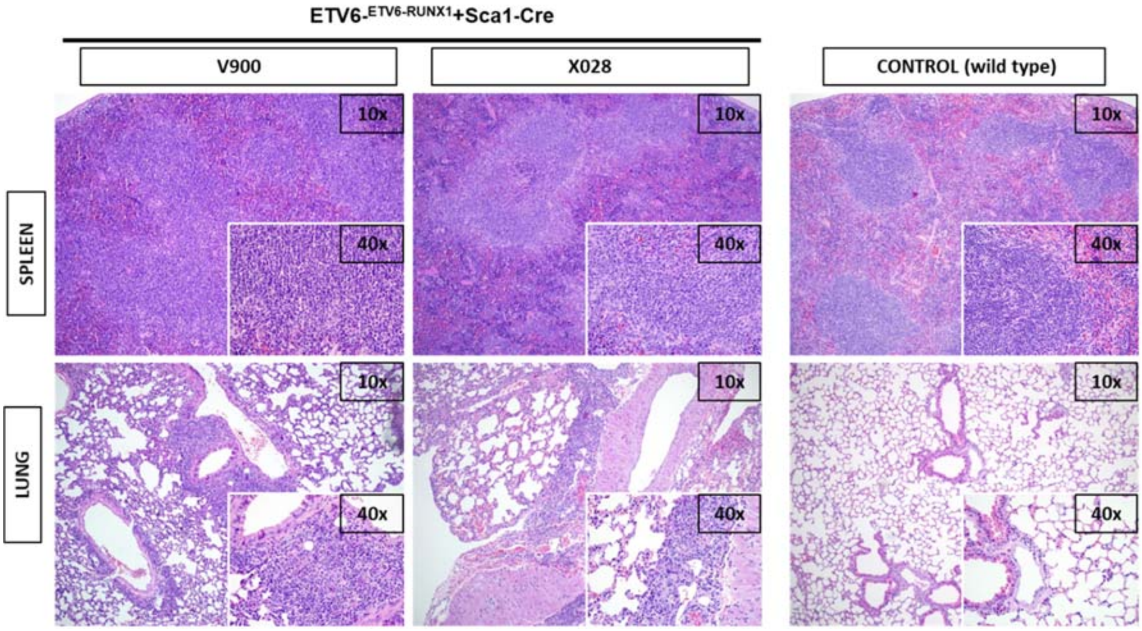

Supplementary Figure 6. Histological images of ETV6-$^{ETV6\text{-}RUNX1}$+Sca1-Cre disease mice with B-ALL. Hematoxylin and eosin staining of spleen and lung from wild-type and tumor-bearing ETV6-$^{ETV6\text{-}RUNX1}$+Sca1-Cre mice shows loss of normal architecture resulting from effacement with cells morphologically resembling lymphoblasts.

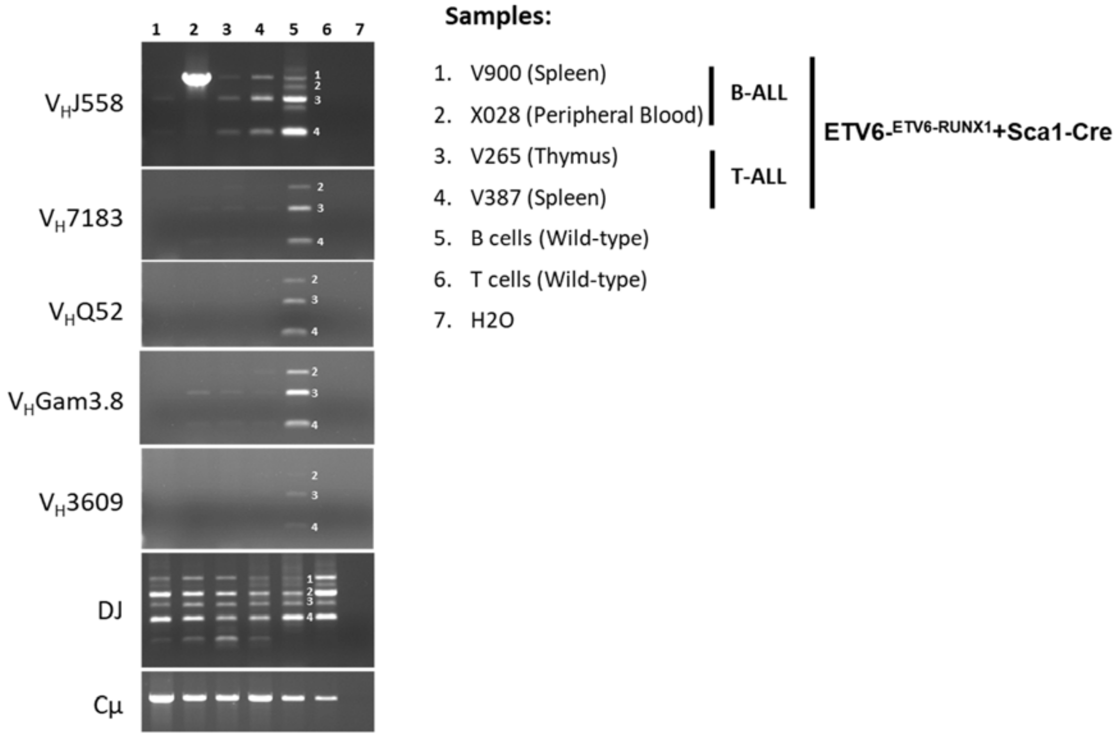

Supplementary Figure 7. BCR clonality in ETV6-$^{ETV6\text{-}RUNX1}$+Sca1-Cre mice with either B-ALL or T-ALL. PCR analysis of BCR gene rearrangements in infiltrated tissues (spleen, peripheral blood, thymus and spleen) of diseased mice. Sorted CD19+ splenic B cells (B cells) of healthy mice serve as a control for polyclonal BCR rearrangements. DP T cells from the thymus of healthy mice served as a negative control. Infiltrated tissues shows an increased clonality within their BCR repertoire (indicated by the code number of each mouse analyzed).

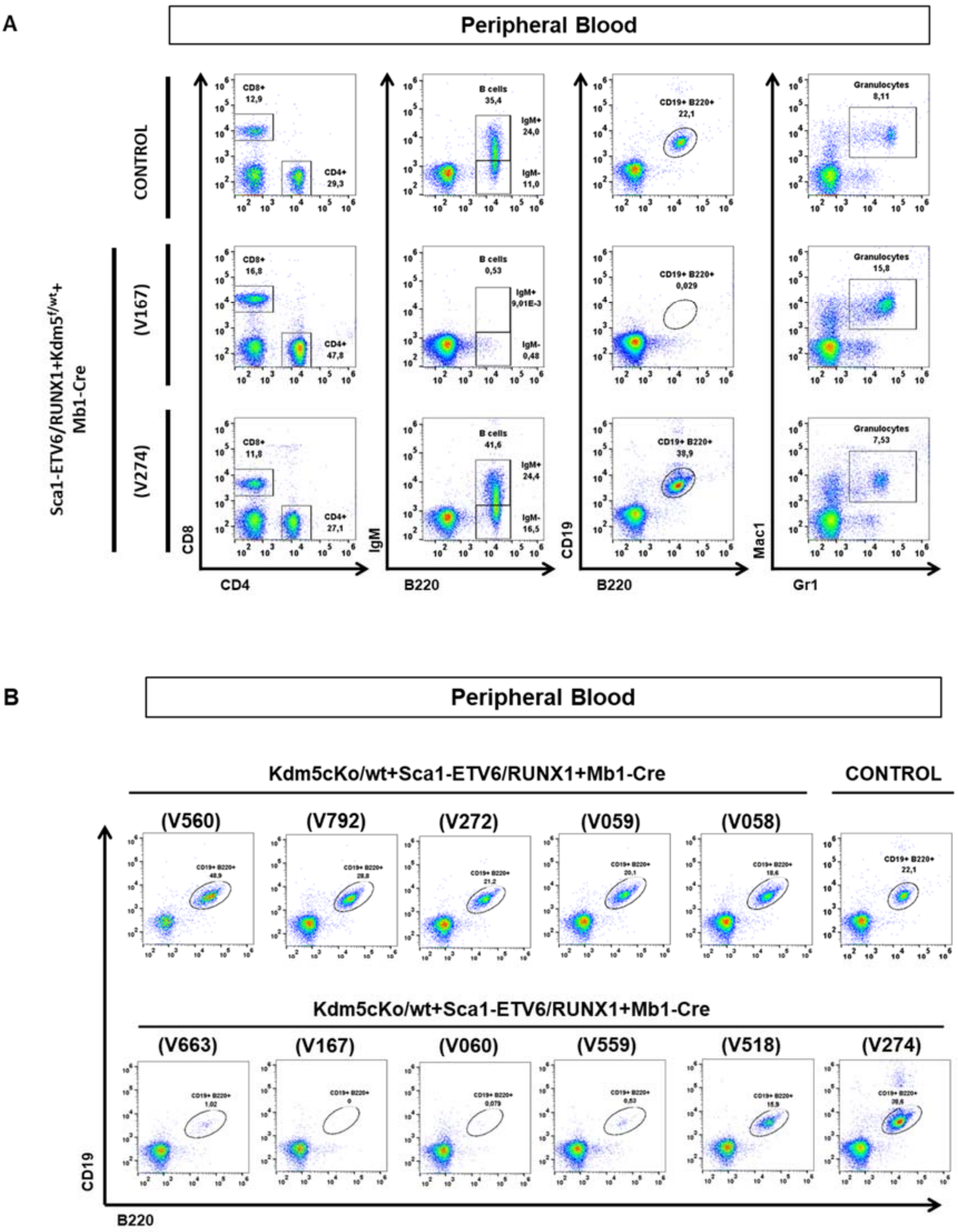

Supplementary Figure 8. The absence of Kdm5c at proB cell level damages B-cell development. A) Representative peripheral blood FACs analysis of distinct cell subsets in Kdm5c$^{f/wt}$+ Sca1-ETV6/RUNX1+Mb1-Cre with (V274) and without (V167) B cells. **B)** B cells (CD19+ B220+) flow cytometry charts of Kdm5c$^{f/wt}$+ Sca1-ETV6/RUNX1+Mb1-Cre mice illustrating the absence of B cells in some of the mice (V663, V167, V060, and V559). An age-matched control wild-type mouse is shown as the control (6 months old).

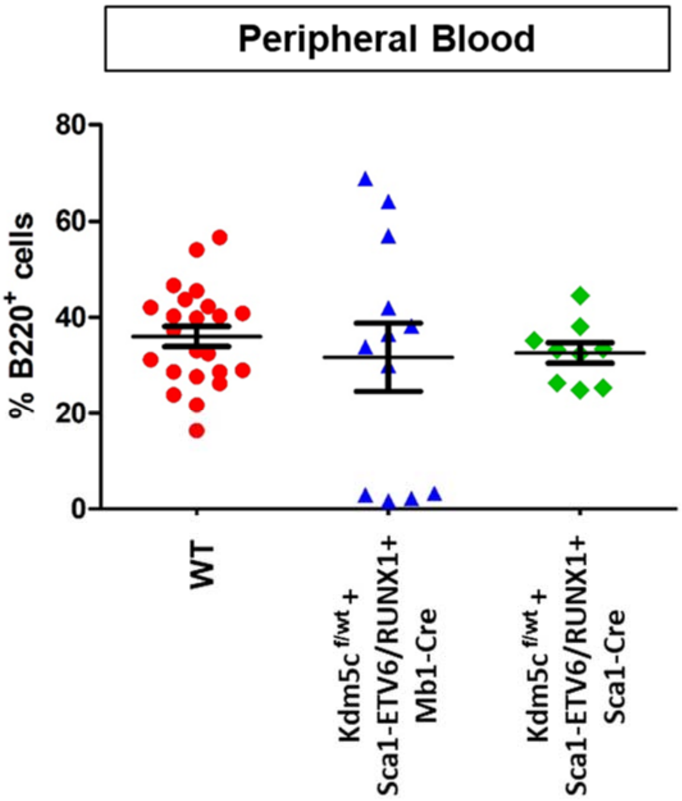

Supplementary Figure 9. HSC-cell specific Kdm5c loss-of-function did not affect B-cell development in Sca1-ETV6-RUNX1+ Kdm5c$^{f/wt}$+Sca1-Cre mice. PB cytometric analysis of B cells from Kdm5c $^{f/wt}$ +Sca1-ETV6/RUNX1+ Sca1-Cre mice (n=9) compared with age-matched Kdm5c $^{f/wt}$ +Sca1-ETV6/RUNX1+ Mb1-Cre (n=11) and age-matched (6 months-old) control wild type mice (n= 23). The unpaired t-test p-values were not significant in any of the potential comparisons. Error bars represent the SD.

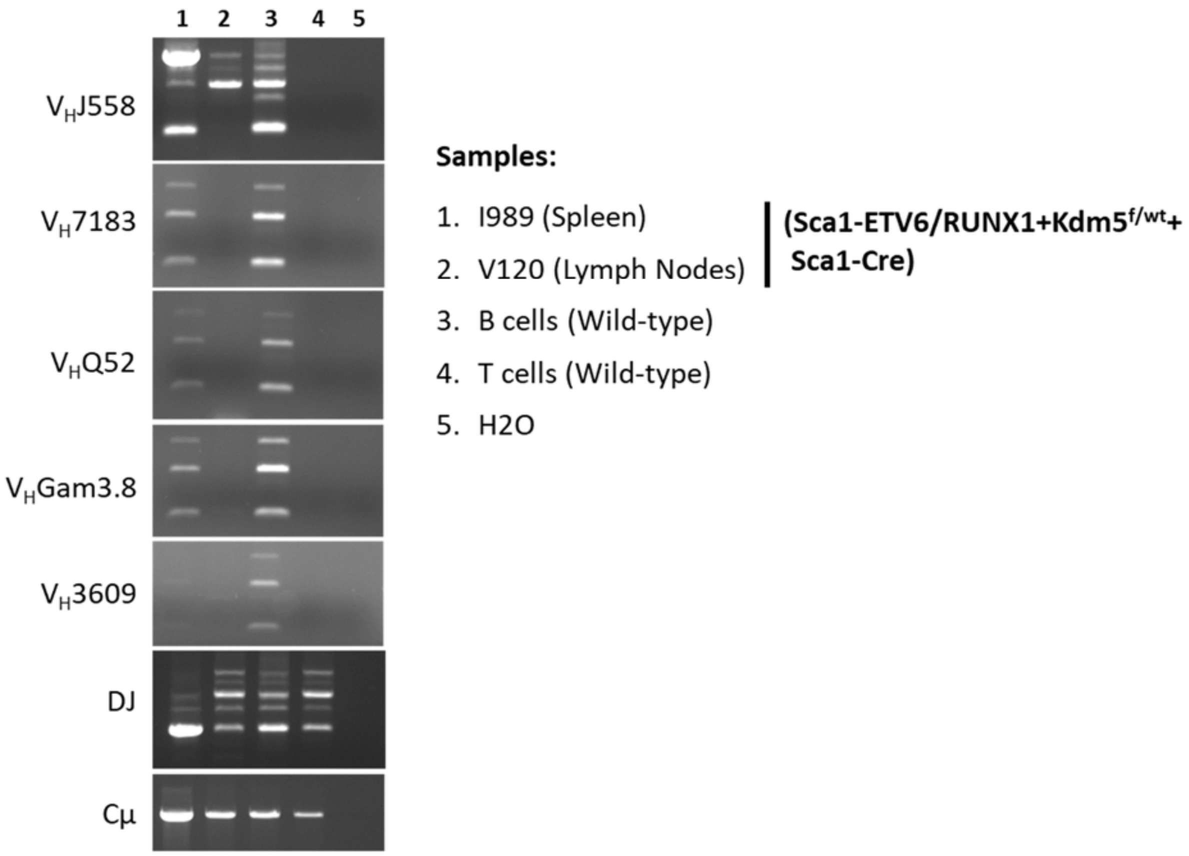

Supplementary Figure 10. **BCR clonality in Sca1-*ETV6-RUNX1*+ Kdm5c$^{f/wt}$+Sca1-Cre mice with B-ALL.** PCR analysis of BCR gene rearrangements in infiltrated tissues (spleen and lymph nodes) of diseased mice. Sorted CD19^{+} splenic B cells (B cells) of healthy mice serve as a control for polyclonal BCR rearrangements. DP T cells from the thymus of healthy mice served as a negative control. Infiltrated tissues shows an increased clonality within their BCR repertoire (indicated by the code number of each mouse analyzed).

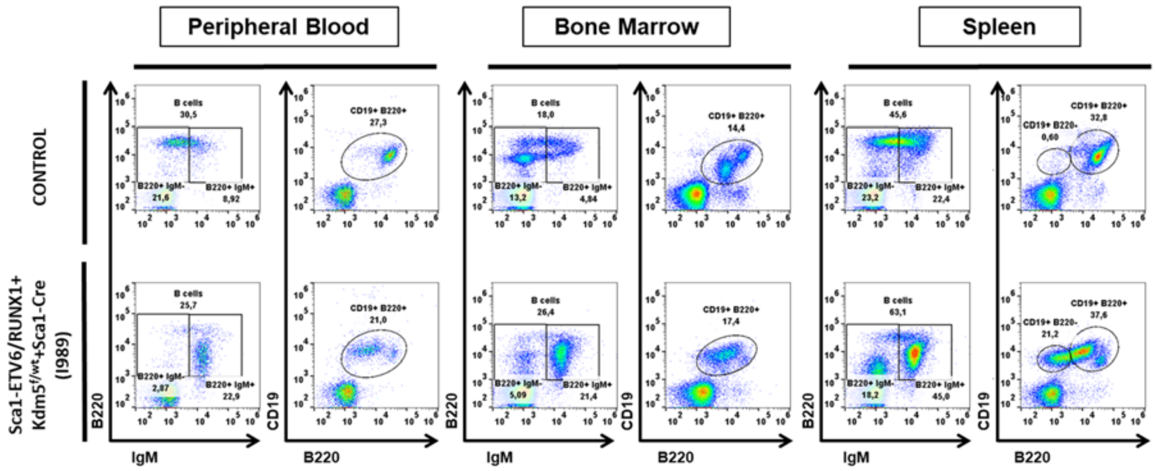

Supplementary Figure 11. B cell neoplasia in Sca1-*ETV6/RUNX1*+ Kdm5c$^{f/wt}$ + Sca1-Cre mice. Flow cytometric analysis of hematopoietic subsets in diseased Sca1-*ETV6/RUNX1*+ Kdm5c$^{f/wt}$ + Sca1-Cre mice. Representative plots of cell subsets from the peripheral blood, bone marrow and spleen show accumulation of blast B cells in disease mice compared to control littermate wild-type mice age-matched (age: 22 months-old).

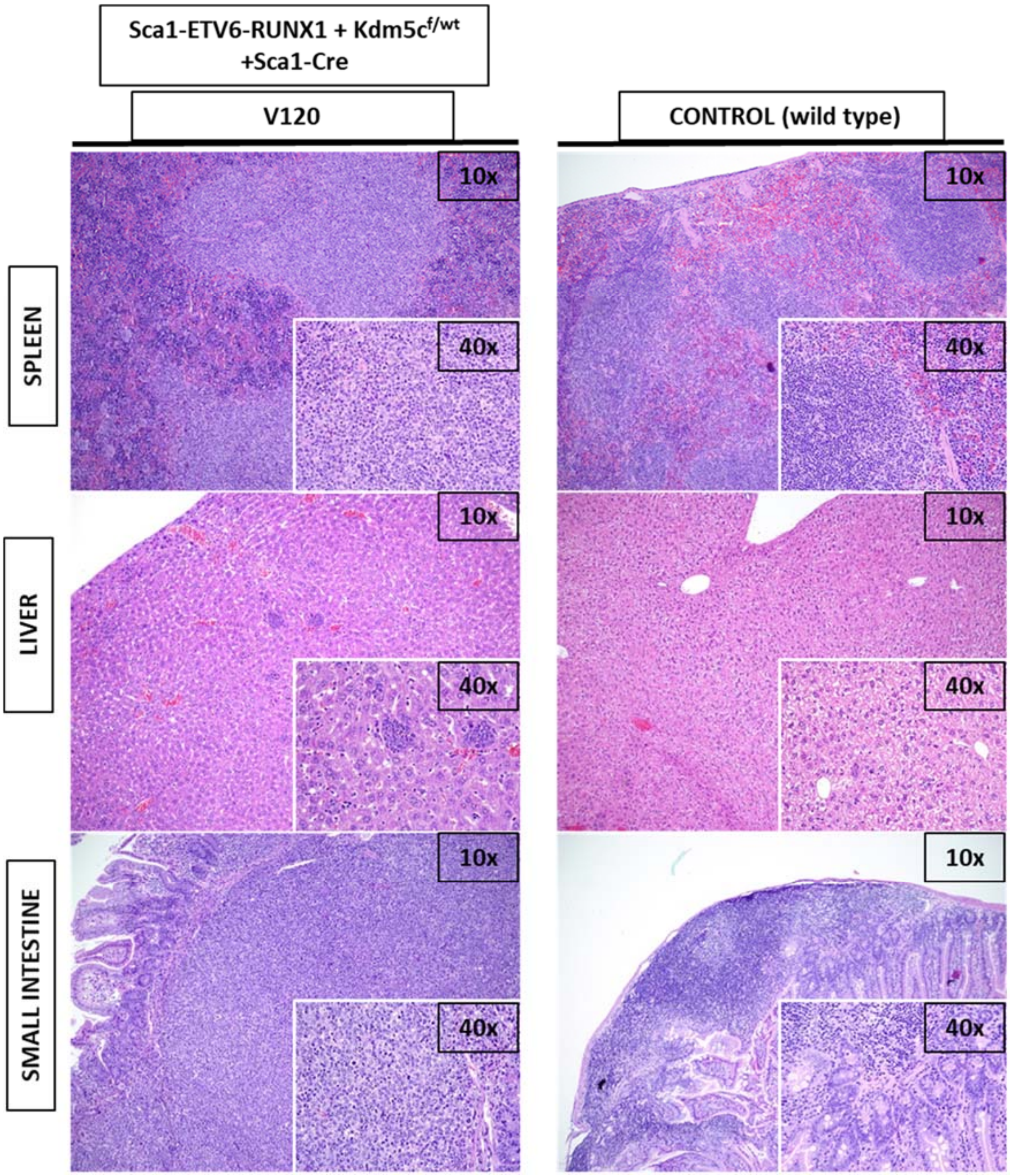

Supplementary Figure 12. Histological images of Sca1-*ETV6/RUNX1*+ Kdm5c$^{f/wt}$ + Sca1-Cre disease mice with B-ALL. Hematoxylin and eosin staining of spleen, liver and small intestine from wild-type and tumor-bearing Sca1-*ETV6/RUNX1*+ Kdm5c$^{f/wt}$ + Sca1-Cre mice shows loss of normal architecture resulting from effacement with tumoral B cells.

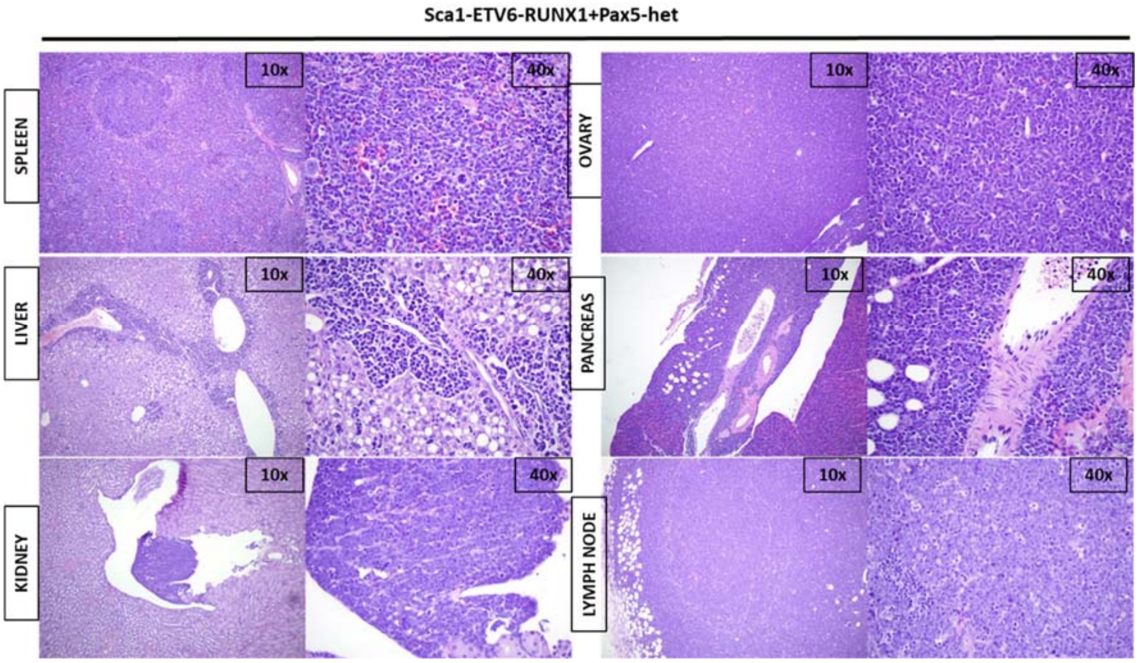

Supplementary Figure 13. Histological images of Sca1-*ETV6/RUNX1*+ Pax5-het disease mice with B-ALL. Hematoxylin and eosin staining of spleen, liver and kidney from tumor-bearing Sca1-*ETV6/RUNX1*+ Pax5-het mice shows loss of normal architecture resulting from effacement with tumoral B cells.

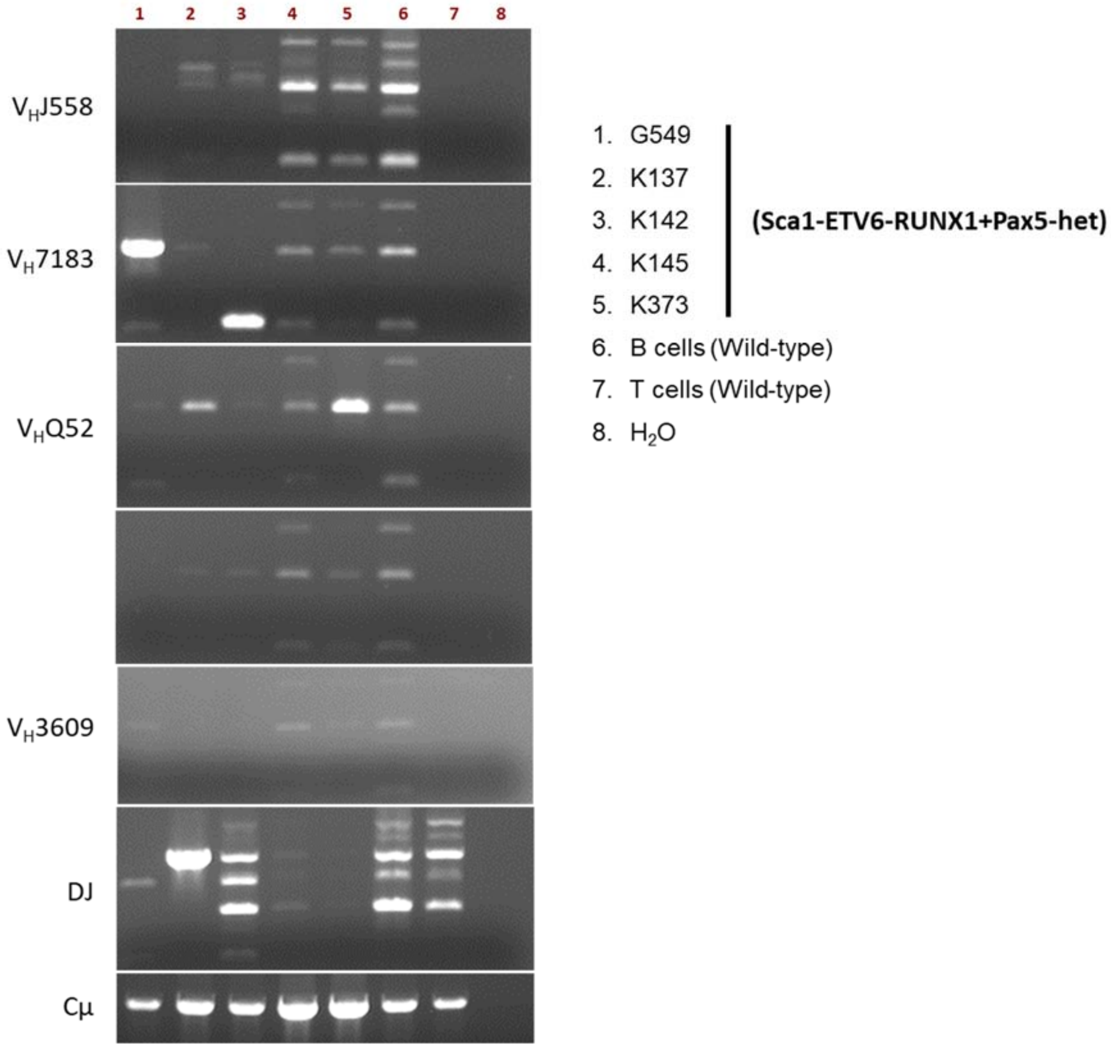

Supplementary Figure 14. BCR clonality in Sca1-*ETV6-RUNX1*+Pax5-het mice with B-ALL. PCR analysis of *BCR* gene rearrangements in infiltrated BM of diseased mice. Sorted CD19$^+$ splenic B cells (B cells) of healthy mice serve as a control for polyclonal BCR rearrangements. DP T cells from the thymus of healthy mice served as a negative control. Infiltrated tissues shows an increased clonality within their BCR repertoire (indicated by the code number of each mouse analyzed).

<u>Checklist S1:</u>

NOTE: Please save this file locally before filling in the table, DO NOT work on the file within your internet browser as changes will not be saved. Adobe Acrobat Reader (available free here) is recommended for completion.

The ARRIVE guidelines 2.0: author checklist

The ARRIVE Essential 10

These items are the basic minimum to include in a manuscript. Without this information, readers and reviewers cannot assess the reliability of the findings.

Item		Recommendation	Section/line number, or reason for not reporting
**Study design**	1	For each experiment, provide brief details of study design including:	
		a. The groups being compared, including control groups. If no control group has been used, the rationale should be stated.	Methods: Paragraph 1<br>Results: Paragraph 1, 2, 3, 4, and 7.
		b. The experimental unit (e.g. a single animal, litter, or cage of animals).	Methods: Paragraph 1<br>Results: Paragraph 1, 2, 3, and 5.
**Sample size**	2	a. Specify the exact number of experimental units allocated to each group, and the total number in each experiment. Also indicate the total number of animals used.	Results: Paragraph 1, 2, 3, and 5.
		b. Explain how the sample size was decided. Provide details of any *a priori* sample size calculation, if done.	Methods: Paragraph 1
**Inclusion and exclusion criteria**	3	a. Describe any criteria used for including and excluding animals (or experimental units) during the experiment, and data points during the analysis. Specify if these criteria were established *a priori*. If no criteria were set, state this explicitly.	Methods: Paragraph 1
		b. For each experimental group, report any animals, experimental units or data points not included in the analysis and explain why. If there were no exclusions, state so.	Methods: Paragraph 1
		c. For each analysis, report the exact value of *n* in each experimental group.	Results: Paragraph 1, 2, 3, and 5.<br>Figures: 2, 3, 5, 6 and 7.<br>Supplementary figures: 9
**Randomisation**	4	a. State whether randomisation was used to allocate experimental units to control and treatment groups. If done, provide the method used to generate the randomisation sequence.	Methods: Paragraph 1
		b. Describe the strategy used to minimise potential confounders such as the order of treatments and measurements, or animal/cage location. If confounders were not controlled, state this explicitly.	Methods: Paragraph 1
**Blinding**	5	Describe who was aware of the group allocation at the different stages of the experiment (during the allocation, the conduct of the experiment, the outcome assessment, and the data analysis).	Methods: Paragraph 1
**Outcome measures**	6	a. Clearly define all outcome measures assessed (e.g. cell death, molecular markers, or behavioural changes).	Methods: Paragraph 1, 2
		b. For hypothesis-testing studies, specify the primary outcome measure, i.e. the outcome measure that was used to determine the sample size.	Methods: Paragraph 1
**Statistical methods**	7	a. Provide details of the statistical methods used for each analysis, including software used.	Methods: Paragraph 16<br>Supplementary Table 3
		b. Describe any methods used to assess whether the data met the assumptions of the statistical approach, and what was done if the assumptions were not met.	Methods: Paragraph 16
**Experimental animals**	8	a. Provide species-appropriate details of the animals used, including species, strain and substrain, sex, age or developmental stage, and, if relevant, weight.	Methods: Paragraphs 1-2
		b. Provide further relevant information on the provenance of animals, health/immune status, genetic modification status, genotype, and any previous procedures.	Methods: Paragraphs 1-2
**Experimental procedures**	9	For each experimental group, including controls, describe the procedures in enough detail to allow others to replicate them, including:	
		a. What was done, how it was done and what was used.	Methods: Paragraphs 1-2-4-6
		b. When and how often.	Methods: Paragraphs 1-2
		c. Where (including detail of any acclimatisation periods).	Methods: Paragraphs 1-2
		d. Why (provide rationale for procedures).	Methods: Paragraphs 2<br>Results: Paragraph 1, 2, 3, 4, and 7.
**Results**	10	For each experiment conducted, including independent replications, report:	
		a. Summary/descriptive statistics for each experimental group, with a measure of variability where applicable (e.g. mean and SD, or median and range).	Figures: 2, 5, and 6.<br>Supplementary figures: 9
		b. If applicable, the effect size with a confidence interval.	Not applicable
